# Rapamycin, Not Metformin, Mirrors Dietary Restriction‐Driven Lifespan Extension in Vertebrates: A Meta‐Analysis

**DOI:** 10.1111/acel.70131

**Published:** 2025-06-18

**Authors:** Edward R. Ivimey‐Cook, Zahida Sultanova, Alexei A. Maklakov

**Affiliations:** ^1^ School of Biodiversity, One Health, and Veterinary Medicine University of Glasgow Glasgow UK; ^2^ School of Biological Sciences University of East Anglia Norwich UK

**Keywords:** dietary restriction, lifespan extension, meta‐analysis, metformin, rapamycin, vertebrate

## Abstract

Dietary restriction (DR) robustly increases lifespan across taxa. However, in humans, long‐term DR is difficult to maintain, leading to the search for compounds that regulate metabolism and increase lifespan without reducing caloric intake. The magnitude of lifespan extension from two such compounds, rapamycin and metformin, remains inconclusive, particularly in vertebrates. Here, we conducted a meta‐analysis comparing lifespan extension conferred by rapamycin and metformin to DR‐mediated lifespan extension across vertebrates. We assessed whether these effects were sex‐ and, when considering DR, treatment‐specific. In total, we analysed 911 effect sizes from 167 papers covering eight different vertebrate species. We find that DR robustly extends lifespan across log‐response means and medians and, importantly, rapamycin—but not metformin—produced a significant lifespan extension. We also observed no consistent effect of sex across all treatments and log‐response measures. Furthermore, we found that the effect of DR was robust to differences in the type of DR methodology used. However, high heterogeneity and significant publication bias influenced results across all treatments. Additionally, results were sensitive to how lifespan was reported, although some consistent patterns still emerged. Overall, this study suggests that rapamycin and DR confer comparable lifespan extension across a broad range of vertebrates.

## Graphical abstract

The authors provide evidence that, together with Dietary Restriction, Rapamcyin and not Metformin, provides a significant lifespan extension in vertebrates.

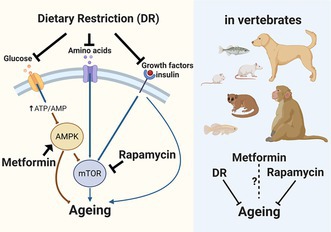

## 1 Introduction

Dietary restriction (DR) is a classical approach to lifespan extension through the reduction of food intake without entering a malnourished state. DR and its lifespan‐extending effects have been the source of study for over 100 years (Osborne et al. 1917; McCay et al. 1935; Selman 2014; although see also Speakman and Mitchell 2011) and have been shown to robustly increase the lifespan of numerous different taxonomic groups, from invertebrate species, such as nematode worms ( *Caenorhabditis elegans* ) or fruit flies ( *Drosophila melanogaster* ), to vertebrate species, such as mice and primates (Bodkin et al. 2003; Anderson et al. 2009; Fontana et al. 2010; see Nakagawa et al. 2012 for a previous meta‐analysis on lifespan extension across model and non‐model organisms). Despite this, the effects appear to not always be universally positive (Harper et al. 2006; Sohal et al. 2009) and in humans, such an imposed and long‐term reduction in caloric intake is often associated with low adherence (Scheen 2008; Barte et al. 2010; Selman 2014; Di Francesco et al. 2024). As a result, substances that mimic a DR response without the need for an active reduction in caloric intake, called DR mimetics, have been put forward as possible alternatives (Mattson et al. 2001; Ingram et al. 2006; Mouchiroud et al. 2010).

Two of the most widely used compounds that have been the focus of much research on lifespan extension to date are rapamycin and metformin. Rapamycin (or Sirolimus) was identified and isolated from Easter Island soil bacteria in 1975 (Vézina et al. 1975) and has been used primarily as an food and drug administation‐approved immunosuppressant for kidney transplants and cardiac stents (Kaeberlein et al. 2023). It is an inhibitor of the mechanistic target of rapamycin (mTOR) pathway and has been shown to extend lifespan and reduce epigenetic ageing across a wide variety of organisms in a manner similar to DR (Harrison et al. 2009; Miller et al. 2011; Swindell 2017; Horvath et al. 2019). Rapamycin has also been found to have a number of benefits in reducing age‐related diseases in humans (Lee et al. 2024). However, in some species, this positive effect is not present, for instance on epigenetic ageing in the common marmoset (Horvath et al. 2021) or rates of ageing in mice (Neff et al. 2013).

The second popular DR mimetic, Metformin (or dimethylbiguanide) is used to combat type II diabetes as it reduces levels of circulating glucose and improves insulin sensitivity in the body (Bailey and Turner 1996). Metformin is an activator of adenosine monophosphate‐activated protein kinase (AMPK) and has been shown to extend lifespan in diverse species, from nematodes (Onken and Driscoll 2010) to mice (Anisimov et al. 2005). It has also been shown to decelerate ageing in male cynomolgus monkeys (Yang et al. 2024). However, the overall effects of metformin on lifespan remain inconclusive (Selman 2014; Mohammed et al. 2021). This highlights the urgent need to (1) reassess the degree to which these two DR mimetics promote a lifespan extension and (2) compare the effects of these two compounds with that of DR. Focusing on these two questions in vertebrate species will allow us to conclusively state which of these two mimetics has the greatest potential as a substitute for long‐term DR in humans.

To this end, we performed a systematic review and meta‐analysis to assess the degree of lifespan extension in vertebrate species under three well‐established longevity treatments: DR (two different types of DR, fasting and caloric reduction) and two well‐known DR‐mimetics, metformin and rapamycin. We also tested two other important moderators: (1) the sex of the animals subjected to each treatment to assess whether the effects were sex‐specific and (2) for DR specifically, the form of methodology used to test whether DR‐specific lifespan extension was sensitive to how DR was implemented.

## 2 Methods

Note, where appropriate we follow MERIT guidelines as per Nakagawa, Ivimey‐Cook, et al. (2023). All data and code are available from Zenodo 10.5281/zenodo.15673918.

### 2.1 Search and Screening

EIC performed a systematic literature search following Preferred Reporting Items for Systematic Reviews and Meta‐Analyses (PRISMA; see Moher et al. 2011), using the databases Scopus and Web of Science first searched in July 2023 and then updated in December 2024 using the search strings found in Table S1 (N.B. searches included both published and unpublished studies via the Web of Science databases). For the searches from July 2023, EIC and ZS manually screened the papers in Rayyan (Ouzzani et al. 2016). We also included references in our filtering that were not found in the original search but were in five papers that appeared in our search (namely, Everitt et al. 2005; Mair and Dillin 2008; Colman et al. 2014; Speakman et al. 2016; Ingram and de Cabo 2017; Selvarani et al. 2021). For the updated search in December 2024, EIC manually screened papers using the metRscreen application (Ivimey‐Cook 2025) after removing duplicates between the 2023 and 2024 searches using the {synthesisr} v. 0.3.0 package (Westgate and Grames 2020). See Figure S1 for a Prisma diagram of searching, screening and filtering. Furthermore, we followed the PRISMA‐EcoEvo checklist created by O'Dea et al. (2021) (Table S2) and checked our meta‐analysis with the MATES (Meta‐analysis Appraisal Tool for Environmental Sciences) checklist for meta‐analysis reporting quality (Morrison et al. 2025; Table S3). In all cases we chose studies where there was an experimental group (typically a control or a treatment without the lifespan intervention) along with a corresponding treatment group (with the lifespan intervention). We only focused on studies that involved vertebrates, provided a measure of lifespan (either mean, median or present in a survival curve), provided some measure of sample size, standard deviation or standard error (and sample size), or in the particular case of studies with survival curves, had survival curves that crossed 50% for the control and experimental cohorts (see Figure S1).

### 2.2 Data Extraction

If raw data was not available (as in most cases) EIC and ZS extracted mean and median lifespan from all accepted papers. EIC then double‐checked all extracted data. ZS checked the reproducibility of the model code. Mean data was initially favoured; however, upon screening several papers, it became apparent that a large proportion of papers simply provided median values of lifespan or presented data in survival curves (with no raw data archived). As a result, we extracted both. If values were provided in table or text, we extracted these directly from the source paper. However, if survival curves were present, EIC and ZS extracted the median lifespan (where survival curves reached 50%) using WebPlotDigitizer (Rohatgi 2017) and, where suitable (for instance when boxplots were present) using metaDigitise v.1.01 (Pick et al. 2018). Where possible, EIC and ZS also extracted a corresponding standard error or standard deviation (for means), or, if these were unavailable (or were medians), a sample size for the control and treatment groups. Any missing standard deviations were then calculated prior to analysis (see below). If any raw data was present, we directly calculated medians along with mean values and corresponding standard deviations. Note that if raw data was presented separately per sex, we did not combine these to create a ‘mixed’ sex grouping. In addition, if censoring were involved, where possible we excluded those that were censored. Lastly, following Ivimey‐Cook et al. (2023), EIC and ZS recorded all locations of the lifespan data from each source paper.

### 2.3 Moderators

For each paper, EIC and ZS also extracted two different moderators, namely:

1. Treatment (Rapamycin, metformin or DR. In the case of DR we noted whether the form of DR was a reduction in intake, removal of food or fasting, we did not include isocaloric reduction in protein or other macromolecules). In all cases, we included a control group (or a treatment without the lifespan intervention) alongside an experimental group that received the added longevity treatment. We also noted if there were any other environmental variables that were used in the study, for instance, the addition of radiation or use of a disease model of mouse. For the DR group only, we recorded whether the experiment involved a percent reduction in calories or food intake (‘Percent Reduction’) or whether the vertebrate was fasted (meaning simply without food for a period of time; ‘Fasted’). Only in one case did a study explicitly test the effect of reduction in food *and* fasting (‘Percent Reduction and Fasted’).
2. Sex of the studied vertebrate (if no sex was mentioned we assumed that both males and females were combined and classed this as ‘mixed’).

### 2.4 Statistical Analysis

All analyses and visualisation used R v. 4.4.2 (R Core Team 2024). EIC calculated the log‐response ratio of means or medians which were adjusted for small‐moderate sample size bias following Lajeunesse (2015). Then, using the rma.mv function from {metafor} v. 4.6–0 (Viechtbauer 2010), EIC ran two multi‐level different models where each effect size was weighted based on the inverse variance–covariance matrix using different approaches to replace missing standard deviations, all cases and missing cases following Nakagawa, Lagisz, et al. (2023) note we changed the tested distribution to *t* distribution throughout, in addition where appropriate to allow convergence we also changed the optimiser to ‘Nelder–Mead’ using the ‘optim’ optimiser). As there were no qualitative differences were detected between the two methods used to replace missing standard deviations, so we present the results from the ‘all cases’ method here (for overall effect of treatment using missing cases, see Figure S2). As there were no qualitative differences between types of approaches, we present all results using the all‐cases method. All models had the fixed moderator of treatment type, and the random effects of species, paper (to account for non‐independence of effects, as in many cases multiple effect sizes originated from the same paper), and an observation level ID to absorb residual variance (Nakagawa and Santos 2012). We then fit a variety of multi‐level models according to the moderators listed above. Average marginal effects from the {emmeans} v. 1.10.6 package (Lenth et al. 2019) were then displayed either using the {orchaRd} v. 2.0 (Nakagawa et al. 2020; Nakagawa, Lagisz, et al. 2023) or {ggplot2} v. 3.5.1 (Wickham 2011) plotting packages alongside the {gt} v. 0.11.1 table package (Iannone et al. 2025). We present data from the model that combines study means and median values together but also, where appropriate, discuss the separate effects. Lastly, publication bias was tested and adjusted for by fitting a model with the inverse of effective sample size (small‐study bias) and mean‐centred year (time‐lag bias) as covariates (see Nakagawa et al. 2021). Lastly, following the methodology of Nakagawa, Lagisz, et al. (2023), we also performed a Geary test to assess adherence of the log‐response ratio of means to a normal distribution following Lajeunesse (2015). As only five out of all 911 effect sizes (0.5%) failed this test, we present results with these five included.

## 3 Results

### 3.1 Effect Sizes

In total, we extracted 911 effect sizes (*k*) from 167 papers (*n*) (McCay et al. 1935; Kibler and Johnson 1966; Leveille 1972; Kendrick 1973; Drori and Folman 1976; Fernandes et al. 1976, 1997; Merry and Holehan 1979; Weindruch and Walford 1982; Yu et al. 1982, 1985, 2019; Cheney et al. 1983; Davis et al. 1983; Lloyd 1984; Kohno et al. 1985; Weindruch et al. 1986; Horáková et al. 1988; Masoro et al. 1989, 1995; Goodrick et al. 1990; Harris et al. 1990; Snyder et al. 1990; Koizumi et al. 1992; Shimokawa et al. 1993, 2003, 2015; Thurman et al. 1994; Murtagh‐Mark et al. 1995; Sheldon et al. 1995; Willott et al. 1995; Hursting et al. 1997; McCarter et al. 1997; Yoshida et al. 1997; Pugh et al. 1999; Turturro et al. 1999; Lingelbach and McDonald 2000; Sell et al. 2000; Sogawa and Kubo 2000; Wolf et al. 2000; Bartke et al. 2001; Jolly et al. 2001; Kealy et al. 2002; Tanaka et al. 2002; Tsao 2002; Bodkin et al. 2003; Sharp 2003; Dhahbi et al. 2004; Lee et al. 2004; Anisimov, Berstein, et al. 2005, 2011; Anisimov, Egormin, et al. 2005, 2010; Anisimov et al. 2008, 2015; Anisimov, Piskunova, et al. 2010; Anisimov, Zabezhinski, et al. 2010, 2011; Hamadeh et al. 2005; Ikeno et al. 2005; Lawler et al. 2005; Hamadeh and Tarnopolsky 2006; Harper et al. 2006, 2010; Ma et al. 2007; Cai et al. 2008; Chen et al. 2008; Garcia et al. 2008; Inness and Metcalfe 2008; Li et al. 2008, 2017; McDonald et al. 2008; Merry et al. 2008; Pearson et al. 2008; Zha et al. 2008; Arum et al. 2009; Harrison et al. 2009, 1984; Buschemeyer et al. 2010; Flurkey et al. 2010; Liao et al. 2010, 2016; Rikke et al. 2010; Smith et al. 2010; Yamaza et al. 2010; Herranz et al. 2011; Miller et al. 2011, 2014; Aires et al. 2012; Cameron et al. 2012; Comas et al. 2012; Komarova et al. 2012; Mattison et al. 2012; Ramos et al. 2012; Martin‐Montalvo et al. 2013; Neff et al. 2013; Ramsey et al. 2014; Sun et al. 2013; Vera et al. 2013; Chiba et al. 2014; Colman et al. 2014; Fok et al. 2014; Hasty et al. 2014; Khapre et al. 2014; López‐Domínguez et al. 2015; Mercken et al. 2014; Popovich et al. 2014; Zhang et al. 2014; Christy et al. 2015; Hurez et al. 2015; Johnson et al. 2015; Huang et al. 2015; Meissner et al. 2015; Arriola Apelo et al. 2016; Kawai et al. 2016; Koopman et al. 2016; Mitchell et al. 2016, 2019; Patel et al. 2016; Richardson et al. 2016; Sataranatarajan et al. 2016; Strong et al. 2016, 2020; Derous et al. 2017; Felici et al. 2017; Guo et al. 2017; Someya et al. 2017; Wang et al. 2017, 2024; Xie et al. 2017; Deepa et al. 2018; Fang et al. 2018; Pifferi et al. 2018; Prokhorova et al. 2018; Reifsnyder et al. 2018; Correia‐Melo et al. 2019; Yamauchi et al. 2019; Ferrara‐Romeo et al. 2020; Palliyaguru et al. 2020; Parihar et al. 2020, 2021; Pomatto et al. 2020; Wei et al. 2020; Liang et al. 2021; Unnikrishnan et al. 2021; Zhu et al. 2021; Acosta‐Rodríguez et al. 2022; Dhillon et al. 2022; McKay et al. 2022; Reijne et al. 2022; Tibarewal et al. 2022; Zaradzki et al. 2022; Duregon et al. 2023; Tseng et al. 2023; Baghdadi et al. 2024; Di Francesco et al. 2024; Sowers et al. 2024; Vermeij et al. 2024; Merry and Holehan 1981; Blackwell et al. 1995; Fernandes et al. 1997; Berrigan et al. 2002; Turturro et al. 2002; Black et al. 2003; Chiba and Ezaki 2010; Harper et al. 2010; Bhattacharya et al. 2012; Bitto et al. 2016; Mattison et al. 2017; Birkisdóttir et al. 2021; Mitchell et al. 2023; Wang et al. 2024) which comprised 354 means (*n* = 81) and 557 (*n* = 160) medians. Unsurprisingly, DR was the most common effect size of the lifespan‐extending treatments (*k* = 677, *n* = 115) followed by rapamycin (*k* = 188, *n* = 38) and metformin (*k* = 46, *n* = 17). Of these, the most represented species was the mouse (*k* = 787, *n* = 127), followed by the rat (*k* = 83, *n* = 32), the rhesus macaque (*k* = 23, *n* = 4), the dog (*k* = 6, *n* = 2), the redtail killifsh (*k* = 5, *n* = 2), the turquoise killifsh (*k* = 4, *n* = 1), the stickleback (*k* = 2, *n* = 1) and, lastly, the mouse lemur (*k* = 1, *n* = 1). The sex that was most studied was male (*k* = 428, *n* = 114) followed by female (*k* = 380, *n* = 77), with several effect sizes originating from mixed‐sex groups (*k* = 103, *n* = 35). For DR, the most common method was through a percent reduction in caloric intake (*k* = 610, *n* = 103), followed by fasting (*k* = 63, *n* = 18), while a combination of both was far less used (*k* = 4, *n* = 1). Across all dietary treatments (and when looking across all measures, means and medians combined), the total heterogeneity (*I* ^2^; or the total variance both between and within studies; Nakagawa et al. 2023) across effect sizes was very high (96.5%) suggesting high variability or inconsistency among effects (Yang et al. 2023). The effect of study ID or the between‐study heterogeneity was less 38.5% than the effect of observation ID or the within‐study effect 58.0%. Lastly, the species effect explained 0% heterogeneity. All other model heterogeneity is given in the supplementary model outputs. Note in all cases, results are presented in the following order: *p* value; estimate (lower confidence interval, higher confidence interval).

### 3.2 Publication Bias

**FIGURE 1:**
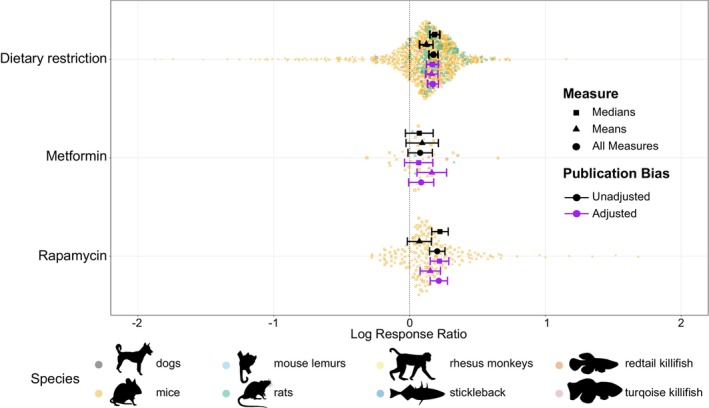
The mean effect of dietary restriction, metformin and rapamycin across vertebrate species. Each treatment has a mean effect size with surrounding 95% confidence intervals. A positive mean effect indicates an overall lifespan‐extending effect of the treatment, whereas a negative is the opposite. Means and errors are shown from models unadjusted (black) or adjusted (purple) for publication bias, as well as originating from models with only medians (squares), only means (triangles) or using both measures combined (circle). Points represent individual effect sizes scaled by precision (1/standard error), shapes denote measure type and colour denotes species (black = dogs, orange = mice, light blue = mouse lemur, green = rats, yellow = rhesus monkeys, dark blue = sticklebacks, dark orange = redtail killifish and pink = turquoise killifish). Silhouettes created using rphylopic v. 1.50 (Gearty and Jones 2023). Attribution: All silhouettes available under creative commons licence CC0 1.0 (dog = Margot Michaud, redtail killifish = Ryan Cupo, turquoise killifish = Tetsuo Kon, rhesus monkey = Ben Murrell, mouse lemur = Arpat Ozgul) and CC BY‐NC‐SA 3.0 (stickleback = Milton Tan). Figure by EIC and ZS.

Overall, there was no evidence of small‐study bias or time‐lag bias influencing the average effect of the longevity treatments across all measures (means and medians combined; *p* = 0.878; −0.018 [−0.242, 0.207] and 0.232, −0.001 [−0.004, 0.001]; Figure 1 and Figure S3). However, when looking at log‐response mean and median values separately, there was significant evidence of small study and time lag bias operating on log‐response means but not medians (indicated by a significant covariate of inverse of effective sample size and mean‐centred year). In particular, small study bias and time‐lag bias were found to be underestimating the overall average effect for each treatment (mean small‐study bias: *p* < 0.001; −0.635 [−0.857, −0.413]; mean time‐lag bias: *p* = 0.011; −0.002 [−0.004, −0.001]). As a result, we interpret results from both measures separately and combined, with and without publication bias adjustment.

### 3.3 Effect of Longevity Treatment

Both the DR and rapamycin treatments were significantly different from zero both with and without adjusting for publication bias in the models when both medians and mean values were combined (with adjustment DR: *p* < 0.001; 0.172 [0.132, 0.213]; with adjustment rapamycin: *p* < 0.001; 0.216 [0.152, 0.279]; without adjustment DR: *p* < 0.001; 0.177 [0.143, 0.210]; without adjustment rapamycin *p* < 0.001; 0.204 [0.147, 0.261]; Figure 1 and S3) but did not differ from each other (with adjustment: *p* = 0.221; 0.044 [−0.026, 0.114]; without adjustment: *p =* 0.406; 0.028 [−0.038, 0.093]; Figure 1 and S3), despite rapamycin having a consistently greater average lifespan extension compared to DR. This suggests that these two treatments produced similar degrees of lifespan extension across all measures. In contrast, the metformin treatment overlapped zero in both models (with adjustment: *p* = 0.069, 0.086 [−0.007, 0.178]; without adjustment: *p =* 0.088; 0.078, [−0.012, 0.168]; Figure 1 and S3), suggesting overall weak support for metformin as a drug to extend lifespan in vertebrates. In both models, metformin was significantly different from rapamycin (with adjustment: *p* = 0.017; 0.130 [0.023, 0.237] and without adjustment: *p =* 0.021; 0.126 [0.019, 0.232]; Figure 1 and S3), and from DR when unadjusted from publication bias (with adjustment: *p* = 0.081; 0.086, [−0.011, 0.184] and without adjustment: *p =* 0.044; 0.098 [0.003, 0.194]; Figure 1 and S3). This pattern remained robust when only looking at studies that used mice (the most represented species; Figure S18) and even, for DR, when effect sizes were limited according to the 900‐day rule (Pabis et al. 2024; Figure S18; although note that the number of effect sizes for metformin was significantly reduced), which was suggested in order to increase the robustness of intervention outcomes. However, the log‐ response ratio of means for rapamycin, unadjusted and adjusted for publication bias, overlapped zero when only using individuals that passed the 900‐day rule (Fig. S18).

In all cases, (log‐response means and medians, with and without adjustment for publication bias), DR was found to extend lifespan (means with adjustment: 0.164 [0.118, 0.209]; means without adjustment: 0.124 [0.075, 0.173]; medians with adjustment: 0.168 [0.124, 0.212]; medians without adjustment: 0.186 [0.149, 0.222]; all *p* < 0.001; Figure 1 and S3–S5). The opposite was true for metformin, as only when looking at log‐response means, adjusted for publication bias, did the average effect of metformin not overlap zero (Figure 1 and S3–S5). For rapamycin, a lifespan‐extending effect was apparent when looking overall, as well as log‐response medians (unadjusted and adjusted) and log‐response means adjusted for publication bias (Figure 1 and S3–S5). Using only log‐response means caused both rapamycin and metformin to produce a similar lifespan extension as DR (with adjustment: *p =* 0.796; −0.011 [−0.092, 0.071] and 0.994; 0.0004 [−0.112, 0.113]; and without adjustment: *p =* 0.274; −0.051 [−0.142, 0.040] and 0.627; −0.030 [−0.153, 0.092]; Figure 1 and S4). The average effect of DR was also not significantly different from rapamycin in both models involving medians, adjusted and unadjusted for publication bias (with adjustment: *p =* 0.166; 0.053 [−0.022, 0.127] and without adjustment: *p =* 0.282; 0.039 [−0.032, 0.109] Figure 1 and S5). The effect of dietary restriction was significantly different from metformin when looking at unadjusted log‐response medians but not when adjusted for publication bias (with adjustment: *p* = 0.071; −0.101 [−0.210, 0.009] and without adjustment: *p* = 0.040; ‐0.114 [−0.222, −0.0054]; Fig 1 and S5).

### 3.4 Effect of Sex and Dietary Methodology

**FIGURE 2:**
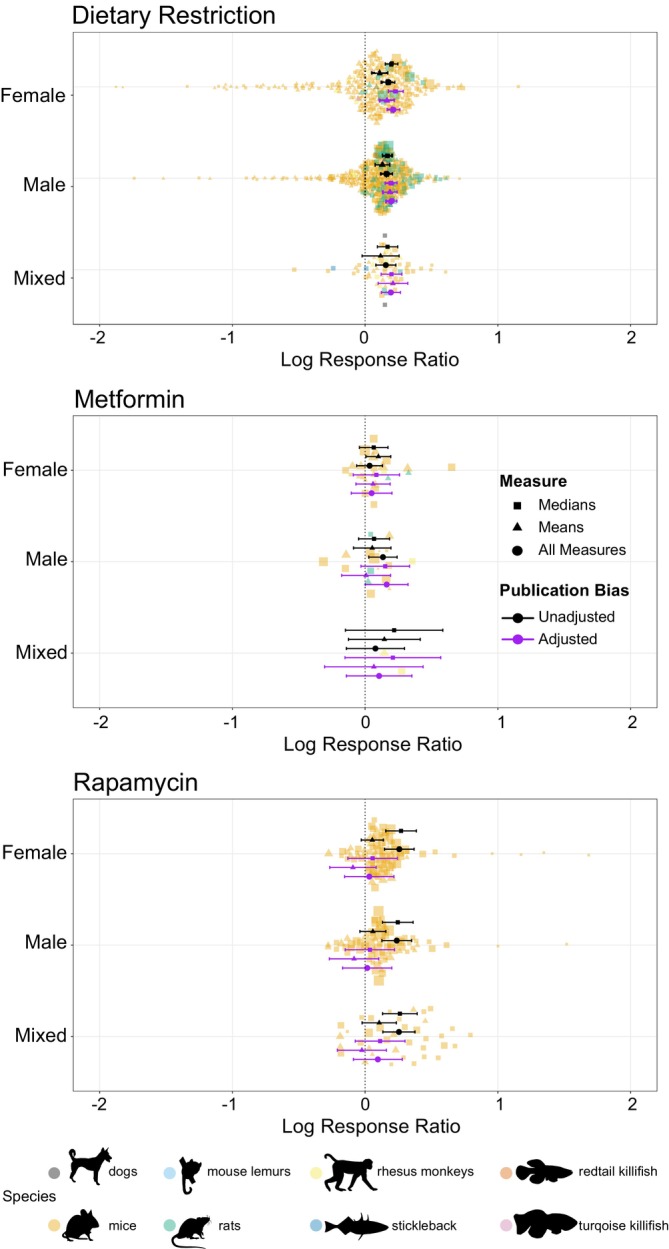
The mean effect of sex under different lifespan‐extension techniques, dietary restriction, metformin and rapamycin across vertebrate species. Each treatment has a mean effect size with surrounding 95% confidence intervals. A positive mean effect indicates an overall lifespan‐extending effect of the treatment, whereas a negative is the opposite. Means and errors are shown from models unadjusted (black) or adjusted (purple) for publication bias, as well as originating from models with only medians (squares), only means (triangles) or using both measures combined (circle). Points represent individual effect sizes scaled by precision (1/standard error), shapes denote measure type and colour denotes species (black = dogs, orange = mice, light blue = mouse lemur, green = rats, yellow = rhesus monkeys, dark blue = stickleback, dark orange = redtail killifish and pink = turquoise killifish). Silhouettes created using rphylopic v. 1.50 (Gearty and Jones 2023), attribution given under Figure 1. Figure by EIC and ZS.

For most models, across all lifespan treatments, the sexes did not significantly differ from each other (Figure 2 and S6–S14). Only in one model for metformin, did publication bias adjusted medians and means combined suggest that males differed significantly from females (*p* = 0.043; 0.113 [0.004, 0.223]).

When testing whether males, females or a combination of both produced a significant lifespan extension, similar variability was found both across treatments and measures. For rapamycin, both adjusted and unadjusted mean values suggested no influence on either sex (adjusted *M*: −0.083 [−0.269, 0.103]; adjusted *F*: −0.092 [−0.266, 0.083]; adjusted Mixed: −0.024 [−0.209, 0.160]; unadjusted *M*: 0.058 [−0.040, 0.156]; unadjusted *F*: 0.054 [−0.290, 0.137]; unadjusted Mixed: 0.106 [−0.023, 0.235]; all *p* > 0.05. Figure 2 and S7). When looking at unadjusted median values, all studied sex groupings were different from zero (unadjusted *M*: 0.246 [0.131, 0.362]; unadjusted *F*: 0.271 [0.155, 0.386]; unadjusted Mixed: 0.262 [0.132, 0.392]; all *p* ≤ 0.001; Figure 2 and S8), which mirrors the overall unadjusted effect with measures combined (unadjusted *M*: 0.238 [0.126, 0.350]; unadjusted *F*: 0.257 [0.146, 0.369]; unadjusted Mixed: 0.255 [0.133, 0.376]; all *p* < 0.001; Figure 2 and S6). After adjusting for publication bias, no sex groupings were different from zero both when looking at log‐response medians and overall (Figure 2 and S6–S8). When looking at metformin, in most circumstances, metformin did not extend the life of either sex (Figure 2 and S9–S11). Only two models, unadjusted means and overall, produced evidence of significant lifespan extension in females (unadjusted means: *p* = 0.038; 0.100 [0.006, 0.193]; Figure 2 and S9,S10) and males (unadjusted overall: *p* = 0.015; 0.134 [0.027, 0.241]; and adjusted overall: *p* = 0.048; 0.162 [0.0014, 0.323]; Fig 2 and S9–10). Once again suggesting weak support for universal lifespan extension in metformin. For DR, a much simpler pattern was observed. Across models with means, medians and both measures combined, both adjusted and unadjusted for publication bias, DR was found to produce a lifespan extension in females, males and mixed sex groupings (Figure 2 and S12–S14). Only when looking at unadjusted mean values was there no lifespan extension in the mixed sex group (*p* = 0.102; 0.117 [−0.023, 0.256]; Figure 2 and S13).

**FIGURE 3:**
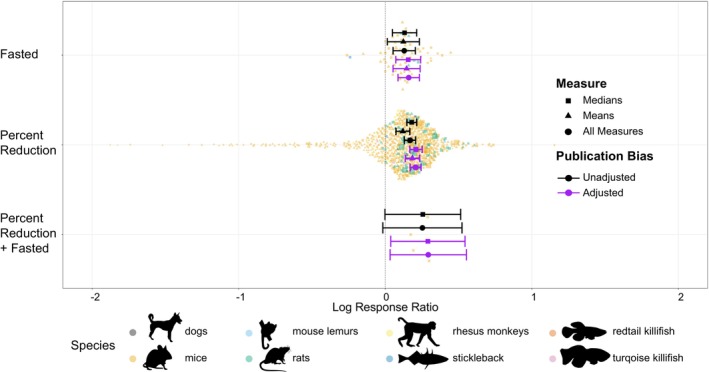
The mean effect of dietary restriction methodologies, Fasting, Percent Reduction and a combination of the two across vertebrate species. Each treatment has a mean effect size with surrounding 95% confidence intervals. A positive mean effect indicates an overall lifespan‐extending effect of the treatment, whereas a negative is the opposite. Means and errors are shown from models unadjusted (black) or adjusted (purple) for publication bias, as well as originating from models with only medians (squares), only means (triangles) or using both measures combined (circle). Points represent individual effect sizes scaled by precision (1/standard error), shapes denote measure type and colour denotes species (black = dogs, orange = mice, light blue = mouse lemur, green = rats, yellow = rhesus monkeys, dark blue = stickleback, dark orange = redtail killifish and pink = turquoise killifish). Silhouettes created using rphylopic v. 1.50 (Gearty and Jones 2023), attribution given under Figure 1. Figure by EIC and ZS.

In addition, both methods of DR with sufficient sample size (percent reduction, and fasting) produced a lifespan extension (Figure 3 and S15–S17). For the singular study which used a method of both, only when measures were adjusted for publication bias did the method produce a significant lifespan extension (although note that this is based on very few effect sizes). However, there were no significant differences between methodologies both overall and when comparing just means or medians adjusted or unadjusted for publication bias (Figure 3 and S15–S17).

## 4 Discussion

The overall aim of this meta‐analysis was to compare the effect of two widely‐studied DR mimetics (rapamycin and metformin) with DR across vertebrates. First, we replicate the general observation found across the animal kingdom that DR promotes robust lifespan extension (Nakagawa et al. 2012) with analogous effects across both males, females and mixed groupings along with no difference in the type of DR methodology employed. Second, we also find compelling evidence that rapamycin, but not metformin, significantly extends lifespan, in most cases similar to that of DR, and that this was robust in mice to the removal of short‐lived controls when looking at medians and overall estimates (Pabis et al. 2024; although note that the log‐response means were not significant). However, we find significant heterogeneity in effects between and within studies as well as, and most notably, we show that lifespan effects can be sensitive to the type of measure reported (i.e., mean vs. median lifespan). Most notably, the positive effect of rapamycin disappears when looking at the log‐response ratio of means, although both metformin and DR appear robust to differences in measure. We also find evidence that publication bias may be obscuring the average effect of these treatments, which after adjusting for small‐study and time‐lag bias, caused the effect of rapamycin to differ significantly from zero in all measures.

**FIGURE 4:**
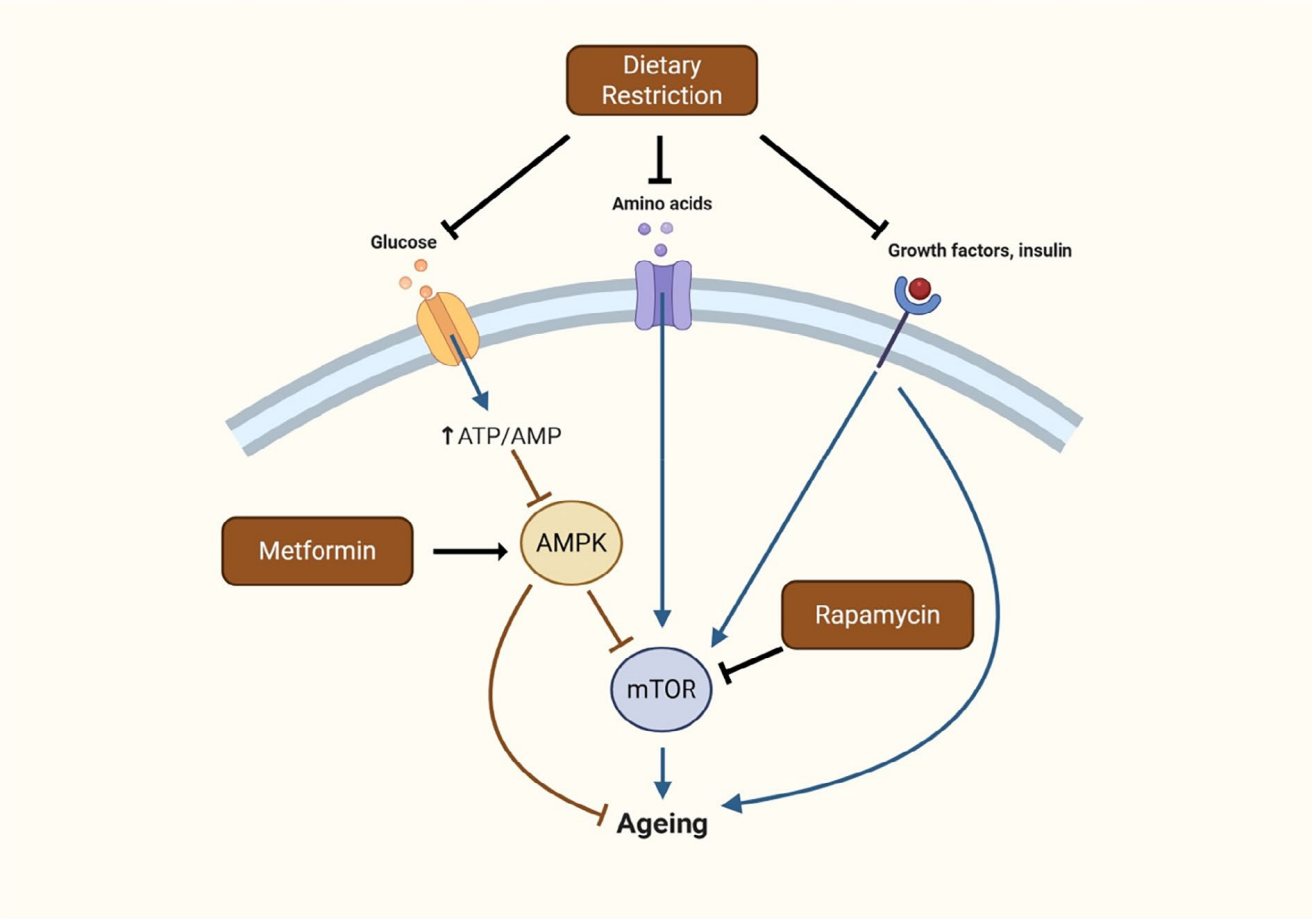
Molecular pathways involved with dietary restriction, metformin or rapamycin. Arrows imply activation; bars denote inhibition. Figure designed by ZS using BioRender.com.

The contrasting effects of rapamycin and metformin (in addition to the robust effect of DR) may in part be due to mechanistic differences in the mediating pathways (Figure 4). Although both DR‐mimetics are classified as mTOR inhibitors, their mode of action is subtly different (Aliper et al. 2017). Whereas rapamycin directly inhibits TOR signalling through the mTORC1 complex, metformin acts indirectly through the activation of the adenosine monophosphate‐activated protein kinase (AMPK), which in turn inhibits TOR signalling (Aliper et al. 2017). Whether a mimetic compound acts directly or indirectly to inhibit TOR signalling may contribute to the differing degrees of lifespan extension reported in this meta‐analysis and, in addition, may explain the added increase in lifespan when both metformin and rapamycin are taken synergistically (Strong et al. 2016; Wolff et al. 2020). Therefore, future work should aim to uncover the precise mechanistic explanation for the observed differences in lifespan extension between these two DR mimetics and how they relate to the various mediating pathways of DR. This is particularly vital as although similar pathways have been identified, the precise mechanisms of action have been shown to differ, particularly between rapamycin and DR (Miller et al. 2014). Finally, DR is known to affect additional pathways beyond AMPK and mTOR, such as growth hormone signalling and insulin/IGF1 signalling pathways, which may explain why DR has more robust effects compared to rapamycin and metformin (Green et al. 2022).

We also explored whether sex was an important modulator of lifespan extension, as previous research had suggested a decreased efficacy of DR in males in comparison to females (Nakagawa et al. 2012). We found no consistent differences in lifespan extension between all sex groupings and across all treatments, although we note the one significant positive effect of males in metformin when accounting for publication bias in combined log‐response means and median. However, overall, the lack of consistent sex effect (particularly in DR) could be due to differences in taxonomic groups studied (across vertebrates and invertebrates in their study and simply vertebrates here) and the calculated effect size (natural log of hazard ratio in their study vs. log‐response means and medians in ours). Nevertheless, we provide evidence of a robust lifespan extension via dietary restriction acting on males, females and mixed sexes. For metformin, as with the general lack of overall effect, there was little evidence of a general sex effect (although note the aforementioned exception), suggesting that regardless of the sex of organism studied, a lifespan extension is unlikely to be found. When observing the effect of sex on rapamycin the results are less clear. Whereas rapamycin had unadjusted median and overall values suggesting an equal lifespan extension acting across all levels of the sex moderator, correcting for publication bias appeared to diminish the positive effect of rapamycin in both sexes. This clearly highlights the need to further assess the sex‐specific efficacy of rapamycin, particularly as the effects have been found to differentially affect males and females across a variety of species across the tree of life (Harrison et al. 2009; Bjedov et al. 2010; Miller et al. 2014; Lind et al. 2016; Raynes et al. 2024).

We also found that the type of DR technique used did not significantly influence the degree of lifespan extension, with two of the main types of DR methodology (percent reduction and fasting) producing a significant extension in lifespan. We note that the third technique, the mixture of both fasting and percent reduction, also produced a significant lifespan extension after adjusting for publication bias. Overall, this is unsurprising as in many cases, aside from the few studies where individuals were withheld from food for prolonged periods, the effects of diet reduction and fasting were often inadvertently entangled. For instance, in several studies, food was restricted to a percentage below *ad libitum* but also with a corresponding reduction to the time period that the subject had to feed (or put another way, increasing the time between feeding periods as typically they were fed only once per day) (see Cheney et al. 1983; Horáková et al. 1988; Black et al. 2003; Chiba and Ezaki 2010; Cameron et al. 2012; Mitchell et al. 2019; Duregon et al. 2021). Only in one study was the reduction in intake and increase in time between feeding explicitly part of the experimental design (Acosta‐Rodríguez et al. 2022). In order to fully distinguish the effects of restricting diet from the effects of fasting, a more appropriate design would be simply to match the timing or duration of feeding of the restricted group with the *ad libitum*, although study subjects may increase feeding rate to compensate for the reduction in calories. However, regardless of the method used, this further highlights the robust lifespan extension that manifests as a result of restricting caloric intake across all studied vertebrate species.

Importantly, we also found that the number of effect sizes originating from median values (*k* = 557) was much larger than from means (*k* = 354). Under a normal distribution, means and median values will be identical; however, medians are often considered a better measure of central tendency than means when data is right‐skewed (frequent low values with a declining number of higher values) or if right censoring has taken place (Bonett and Price 2019), which is often the case for survival data. An obvious easy solution would be for all papers to report both the median and mean survival statistics alongside the provision of raw data in order to more easily conduct meta‐analyses of this type in the future. Whilst not ideal, as median values do not readily provide measures of variance around them, techniques exist to impute missing standard deviations based on existing data (see Nakagawa, Yang, et al. 2023). As a result, simply ignoring median values, which appear to be far more prevalent in literature surrounding DR and related mimetics, risks drawing pre‐emptive conclusions based on a reduced sample of purely log‐response ratio of means. We note that in the log‐response ratio of means, publication bias (here in the form of the moderator of the inverse of effective sample size and mean‐centered year of pulbication) was found to be significantly influencing the reported lifespan extension of all three techniques. Despite this, consistent patterns were observed, namely, DR promoted a robust increase in lifespan across all measures, whereas most measures suggested a significant lifespan extension for rapamycin, and a lack of it for metformin.

Lastly, whilst we provide compelling evidence for the lifespan‐extending efficacy of rapamycin, we emphasise the need for much further research. Firstly, this meta‐analysis was confined to a small number of vertebrate species studied mostly under laboratory conditions. As a result, there is a need for additional studies to explore the generalizability and applicability of these DR mimetics across other vertebrate species, particularly in humans (although early indications of rapamycin and DR appear positive; Aversa et al. 2024; Lee et al. 2024), and in species that can be studied both in the laboratory and in their natural environments. Secondly, there is a need to investigate the heterogeneity in effects that exists across different strains of the same species exposed to the same treatment (Harrison and Archer 1987; Rikke et al. 2010). In particular, why there appears to be large genotype‐specific variation in response to reduced caloric intake or DR mimetics, with some strains showing positive effects while others exhibiting the opposite (Liao et al. 2010; Swindell 2012, 2017). Answering these outstanding questions will provide far deeper insights into the mechanisms and ubiquity of DR‐ or DR‐mimetic‐mediated lifespan extension.

## Author Contributions

E.R.I.‐C. and A.A.M. conceived the study. E.R.I.‐C. and Z.S. contributed to the literature review. E.R.I.‐C. and Z.S. performed data extractions. E.R.I.‐C. performed the data analysis and wrote the manuscript. All authors contributed to revisions and approved the final version of the manuscript.

## Conflicts of Interest

The authors declare no conflicts of interest.

## Supporting information


Data S1.

## Data Availability

Data and code used to reproduce the analyses are available on Zenodo 10.5281/zenodo.15673918.
