# Supplementary material for: Rapamycin, Not Metformin, Mirrors Dietary Restriction‐Driven Lifespan Extension in Vertebrates: A Meta‐Analysis
Source: Aging Cell. 2025 Jun 18;24(9):e70131. doi: 10.1111/acel.70131 (PMC12419861; doi:10.1111/acel.70131)
Supplement: Supplementary file 1 — Data S1. [file ACEL-24-e70131-s001.docx]

**Supplementary material for: Rapamycin, not metformin, mirrors dietary restriction-driven lifespan extension in vertebrates: a meta-analysis**

*Edward R. Ivimey-Cook^1^*, Zahida Sultanova^2*^_,_ and Alexei A. Maklakov^2^*

**Table S1.** Search strings used to search the Scopus and Web of Science databases. Columns denote “AND” rows denote “OR”.

| **Treatment Terms** | **Trait Terms** | **Species Terms** | **Excluded Species (NOT)** |
| --- | --- | --- | --- |
| Rapamycin | Longevity | Vertebrate | Drosophila |
| Metformin | Lifespan | Rat | Elegans |
| Fast* | “Life Expectancy” | Fish | - |
| Dietary Restriction | - | Human | - |
| Caloric Restriction | - | Mouse | - |
| - | - | Mice | - |
| - | - | Bird | - |

Specific search strings WOS: **TS= ((rapamycin OR metformin OR fast* OR "dietary restriction" OR "caloric restriction" ) AND (longevity OR lifespan OR "life expectancy") AND (vertebrate OR rat OR fish OR human OR mouse OR mice OR bird) NOT (drosophila OR elegans))**

Specific search strings Scopus: TITLE-ABS-KEY((rapamycin OR metformin OR fast* OR "dietary restriction" OR "caloric restriction" ) AND (longevity OR lifespan OR "life expectancy") AND (vertebrate OR rat OR fish OR human OR mouse OR mice OR bird) AND NOT (drosophila OR elegans))

**Table S2.** PRISMA Eco-Evo checklist

| **Checklist item** | **Sub-item number** | **Sub-item** | **Reported by authors?** | **Notes** |
| --- | --- | --- | --- | --- |
| **Title and abstract** | 1.1 | Identify the review as a systematic review, meta-analysis, or both | Yes | Title and Abstract |
|  | 1.2 | Summarise the aims and scope of the review | Yes | Abstract |
|  | 1.3 | Describe the data set | Yes | Abstract |
|  | 1.4 | State the results of the primary outcome | Yes | Abstract |
|  | 1.5 | State conclusions | Yes | Abstract |
|  | 1.6 | State limitations | Yes | Abstract and Discussion |
| **Aims and questions** | 2.1 | Provide a rationale for the review | Yes | Introduction |
|  | 2.2 | Reference any previous reviews or meta-analyses on the topic | Yes | Introduction |
|  | 2.3 | State the aims and scope of the review (including its generality) | Yes | Introduction |
|  | 2.4 | State the primary questions the review addresses (e.g. which moderators were tested) | Yes | Introduction |
|  | 2.5 | Describe whether effect sizes were derived from experimental and/or observational comparisons | Yes | In all cases, studies control and treatment groups. Methods. |
| **Review registration** | 3.1 | Register review aims, hypotheses (if applicable), and methods in a time-stamped and publicly accessible archive and provide a link to the registration in the methods section of the manuscript. Ideally registration occurs before the search, but it can be done at any stage before data analysis. | No |  |
|  | 3.2 | Describe deviations from the registered aims and methods | No |  |
|  | 3.3 | Justify deviations from the registered aims and methods | No |  |
| **Eligibility criteria** | 4.1 | Report the specific criteria used for including or excluding studies when screening titles and/or abstracts, and full texts, according to the aims of the systematic review (e.g. study design, taxa, data availability) | Yes | Methods |
|  | 4.2 | Justify criteria, if necessary (i.e. not obvious from aims and scope) | Yes | Methods |
| **Finding studies** | 5.1 | Define the type of search (e.g. comprehensive search, representative sample) | Yes | Methods |
|  | 5.2 | State what sources of information were sought (e.g. published and unpublished studies, personal communications) | Yes | Methods |
|  | 5.3 | Include, for each database searched, the exact search strings used, with keyword combinations and Boolean operators | Yes | Table S1 |
|  | 5.4 | Provide enough information to repeat the equivalent search (if possible), including the timespan covered (start and end dates) | Yes | Methods + Table S1 |
| **Study selection** | 6.1 | Describe how studies were selected for inclusion at each stage of the screening process (e.g. use of decision trees, screening software) | Yes | Methods |
|  | 6.2 | Report the number of people involved and how they contributed (e.g. independent parallel screening) | Yes | Methods |
| **Data collection process** | 7.1 | Describe where in the reports data were collected from (e.g. text or figures) | Yes | Methods |
|  | 7.2 | Describe how data were collected (e.g. software used to digitize figures, external data sources) | Yes | Methods |
|  | 7.3 | Describe moderator variables that were constructed from collected data (e.g. number of generations calculated from years and average generation time) | Yes | Methods |
|  | 7.4 | Report how missing or ambiguous information was dealt with during data collection (e.g. authors of original studies were contacted for missing descriptive statistics, and/or effect sizes were calculated from test statistics) | Yes | Methods |
|  | 7.5 | Report who collected data | Yes | Methods |
|  | 7.6 | State the number of extractions that were checked for accuracy by co-authors | Yes | Methods |
| **Data items** | 8.1 | Describe the key data sought from each study | Yes | Methods |
|  | 8.2 | Describe items that do not appear in the main results, or which could not be extracted due to insufficient information | NA | Methods |
|  | 8.3 | Describe main assumptions or simplifications that were made (e.g. categorising both 'length' and 'mass' as 'morphology') | Yes | Methods |
|  | 8.4 | Describe the type of replication unit (e.g. individuals, broods, study sites) | Yes | Methods |
| **Assessment of individual study quality** | 9.1 | Describe whether the quality of studies included in the systematic review or meta-analysis was assessed (e.g. blinded data collection, reporting quality, experimental versus observational) | No | Study quality was not incorporated |
|  | 9.2 | Describe how information about study quality was incorporated into analyses (e.g. meta-regression and/or sensitivity analysis) | No | Study quality was not incorporated |
| **Effect size measures** | 10.1 | Describe effect size(s) used | Yes | Methods |
|  | 10.2 | Provide a reference to the equation of each calculated effect size (e.g. standardised mean difference, log response ratio) and (if applicable) its sampling variance | Yes | Methods |
|  | 10.3 | If no reference exists, derive the equations for each effect size and state the assumed sampling distribution(s) | NA | Reference exists |
| **Missing data** | 11.1 | Describe any steps taken to deal with missing data during analysis (e.g. imputation, complete case, subset analysis) | Yes | Methods – all cases vs missing cases imputation of variance |
|  | 11.2 | Justify the decisions made to deal with missing data | Yes | Methods |
| **Meta-analytic model description** | 12.1 | Describe the models used for synthesis of effect sizes | Yes | Methods |
|  | 12.2 | The most common approach in ecology and evolution will be a random-effects model, often with a hierarchical/multilevel structure. If other types of models are chosen (e.g. common/fixed effects model, unweighted model), provide justification for this choice | Yes | Methods |
| **Software** | 13.1 | Describe the statistical platform used for inference (e.g. R) | Yes | Methods |
|  | 13.2 | Describe the packages used to run models | Yes | Methods |
|  | 13.3 | Describe the functions used to run models | Yes | Methods |
|  | 13.4 | Describe any arguments that differed from the default settings | Yes | Methods |
|  | 13.5 | Describe the version numbers of all software used | Yes | Methods |
| **Non-independence** | 14.1 | Describe the types of non-independence encountered (e.g. phylogenetic, spatial, multiple measurements over time) | Yes | Methods |
|  | 14.2 | Describe how non-independence has been handled | Yes | Methods |
|  | 14.3 | Justify decisions made | Yes | Methods |
| **Meta-regression and model selection** | 15.1 | Provide a rationale for the inclusion of moderators (covariates) that were evaluated in meta-regression models | Yes | Methods |
|  | 15.2 | Justify the number of parameters estimated in models, in relation to the number of effect sizes and studies (e.g. interaction terms were not included due to insufficient sample sizes) | No | Only univariate analyses took place. |
|  | 15.3 | Describe any process of model selection | NA | No model selection took place |
| **Publication bias and sensitivity analysis** | 16.1 | Describe assessments of the risk of bias due to missing results (e.g. publication, time-lag, and taxonomic biases) | Yes | Methods |
|  | 16.2 | Describe any steps taken to investigate the effects of such biases (if present) | Yes | Methods |
|  | 16.3 | Describe any other analyses of robustness of the results, e.g. due to effect size choice, weighting or analytical model assumptions, inclusion or exclusion of subsets of the data, or the inclusion of alternative moderator variables in meta-regressions | Yes | Results |
| **Clarification of post hoc analyses** | 17.1 | When hypotheses were formulated after data analysis, this should be acknowledged. | NA | No hypotheses were formulated after data analysis |
| **Metadata, data, and code** | 18.1 | Share metadata (i.e. data descriptions) | Yes | Zenodo link in methods |
|  | 18.2 | Share data required to reproduce the results presented in the manuscript | Yes | Zenodo link in methods |
|  | 18.3 | Share additional data, including information that was not presented in the manuscript (e.g. raw data used to calculate effect sizes, descriptions of where data were located in papers) | Yes | Zenodo link in methods |
|  | 18.4 | Share analysis scripts (or, if a software package with graphical user interface (GUI) was used, then describe full model specification and fully specify choices) | Yes | Zenodo link in methods |
| **Results of study selection process** | 19.1 | Report the number of studies screened | Yes | Supplementary |
|  | 19.2 | Report the number of studies excluded at each stage of screening | Yes | Supplementary |
|  | 19.3 | Report brief reasons for exclusion from the full text stage | Yes | Supplementary |
|  | 19.4 | Present a Preferred Reporting Items for Systematic Reviews and Meta-Analyses (PRISMA)-like flowchart (www.prisma-statement.org). | Yes | Supplementary |
| **Sample sizes and study characteristics** | 20.1 | Report the number of studies and effect sizes for data included in meta-analyses | Yes | Results |
|  | 20.2 | Report the number of studies and effect sizes for subsets of data included in meta-regressions | Yes | In graphs in main text + supplementary. |
|  | 20.3 | Provide a summary of key characteristics for reported outcomes (either in text or figures; e.g. one quarter of effect sizes reported for vertebrates and the rest invertebrates) | Yes | Results |
|  | 20.4 | Provide a summary of limitations of included moderators (e.g. collinearity and overlap between moderators) | No | No limitations in included moderators. |
|  | 20.5 | Provide a summary of characteristics related to individual study quality (risk of bias) | NA | The study quality was not investigated here |
| **Meta-analysis** | 21.1 | Provide a quantitative synthesis of results across studies, including estimates for the mean effect size, with confidence/credible intervals | Yes | Results |
| **Heterogeneity** | 22.1 | Report indicators of heterogeneity in the estimated effect (e.g. I2, tau2 and other variance components) | Yes | Results |
| **Meta-regression** | 23.1 | Provide estimates of meta-regression slopes (i.e. regression coefficients) and confidence/credible intervals | Yes | Results or SM |
|  | 23.2 | Include estimates and confidence/credible intervals for all moderator variables that were assessed (i.e. complete reporting) | Yes | Results or SM |
|  | 23.3 | Report interactions, if they were included | NA | No interactions |
|  | 23.4 | Describe outcomes from model selection, if done (e.g. R2 and AIC) | NA | No model selection took place |
| **Outcomes of publication bias and sensitivity analysis** | 24.1 | Provide results for the assessments of the risks of bias (e.g. Egger's regression, funnel plots) | Yes | Results |
|  | 24.2 | Provide results for the robustness of the review's results (e.g. subgroup analyses, meta-regression of study quality, results from alternative methods of analysis, and temporal trends) | Yes | Results |
| **Discussion** | 25.1 | Summarise the main findings in terms of the magnitude of effect | Yes | Discussion |
|  | 25.2 | Summarise the main findings in terms of the precision of effects (e.g. size of confidence intervals, statistical significance) | Yes | Discussion |
|  | 25.3 | Summarise the main findings in terms of their heterogeneity | Yes | Discussion |
|  | 25.4 | Summarise the main findings in terms of their biological/practical relevance | Yes | Discussion |
|  | 25.5 | Compare results with previous reviews on the topic, if available | Yes | Discussion |
|  | 25.6 | Consider limitations and their influence on the generality of conclusions, such as gaps in the available evidence (e.g. taxonomic and geographical research biases) | Yes | Discussion |
| **Contributions and funding** | 26.1 | Provide names, affiliations, and funding sources of all co-authors | Yes | Title page + Acknowledgments |
|  | 26.2 | List the contributions of each co-author | Yes | Contributions statement |
|  | 26.3 | Provide contact details for the corresponding author | Yes | Title page |
|  | 26.4 | Disclose any conflicts of interest | Yes | COI statement |
| **References** | 27.1 | Provide a reference list of all studies included in the systematic review or meta-analysis | Yes | Results |
|  | 27.2 | List included studies as referenced sources (e.g. rather than listing them in a table or supplement) | Yes | Results |

**Table S3.** Meta-analysis Appraisal Tool for Environmental Sciences (MATES) checklist (see Morrison et al., 2025). Columns correspond to the MATES item, the response relating to this paper, and the comment regarding the item.

| **MATES Item** | **Response** | **Comment** |
| --- | --- | --- |
| MATES item 1: Effect size statistic formula reporting | Yes | lnRR (of means or medians) with small sample correction as per Lajeunesse (2015) - Methods |
| MATES item 2: Model type reporting | Yes | Multi-level models – Methods. |
| MATES item 3: Effect size dependence (non-independence)  reporting | Yes | Addition of paper random effect to account for non-independence – Methods. |
| MATES item 4: Software/programming language reporting | Yes | R v. 4.4.2 (R Core Team, 2024) + all associated packages and versions – Methods and in Zenodo sessioninfo.txt. |
| MATES item 5: Number of included primary studies reporting | Yes | 911 effect sizes (k) from 167 papers (n) - Results + Prisma diagram in SM. |
| MATES item 6: Number of included effect sizes reporting | Yes | 911 effect sizes (k) from 167 papers (n) - Results + Prisma diagram in SM. |
| MATES item 7: Pooled mean effect reporting | Yes | The mean effect of each treatment – Results. |
| MATES item 8: Effect size weighting strategy reporting | Yes | inverse variance-covariance matrix – Methods. |
| MATES item 9: Heterogeneity reporting | Yes | Overall heterogeneity intercept-only model – Results. |
| MATES item 10: Publication bias reporting | Yes | Multiple statistical and graphical assessments throughout the paper – Results. |
| MATES item 11: Sensitivity analysis reporting | Yes | In addition to the publication bias correction applied to each subset analysis. We tested each result using a response ratio of means, medians, and all together to check the robustness of the results. Lastly we also used two different methods to fill missing standard deviations (missing cases and all cases) in the models of overall treatment – these did not produce qualitatively different results. Methods + Results. |
| MATES item 12: Code reporting | Yes | Code is deposited in a repository with a link in the Methods. |
| MATES item 13: Data reporting/archiving | Yes | Data used to calculate effect sizes is also deposited in the repository. |
| MATES item 14: Variable description reporting | Yes | Metadata provided with the data in the repository |

Rejected Articles

Total n = 14531

Update search December:

Scopus = 9067

WoS = 12717

Scopus

(1946-2025)

n = 8191

Web of Science

(1913-2025)

n = 8707

Preliminary Screening (Post-Duplicates)

n = 15017

Full Text Screening

n = 480

Accepted Articles

n = 167

Rejected Articles

Total n = 313

No Lifespan Data = 82

Can’t find paper = 51

Can’t access = 28

Wrong study system = 3

Not DR = 9

Duplicate = 8

Retracted = 1

No control = 4

Unclear or missing sample size = 18

Not Relevant = 15

Unclear lifespan data = 4

Wrong/Unclear treatments = 14

Review or contains published data = 53

Treatments not reached median = 23

**Figure S1.** Preferred Reporting Items for Systematic Reviews and Meta-Analyses (PRISMA) diagram detailing the screening and filtering process from initial database searches to accepted articles (first search 27^th^ July 2023. Update search performed 16^th^ December 2024).

**
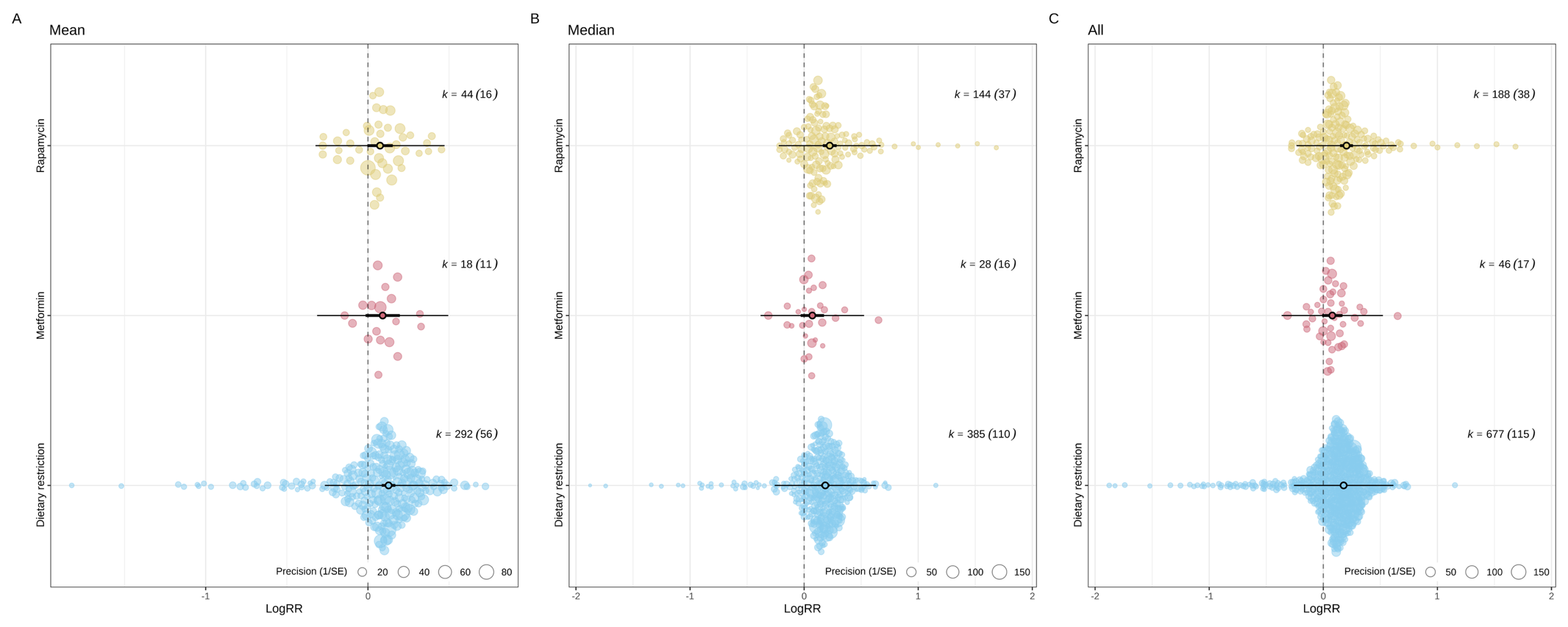
Figure S2.** Orchard plots of the mean effect sizes of dietary restriction (blue), metformin (red), and rapamycin (yellow) across vertebrate species. Showing the mean effect sizes of means (A), medians (B), and all measures (C) calculated using the missing cases method of calculating missing standard deviation. The coloured dots represent individuals effects sized by precision (1/standard error). Each treatment has a mean effect size with surrounding 95% confidence intervals (larger lines) and prediction intervals (thinner lines). The number of effect sizes is given on the right with studies in brackets.

**
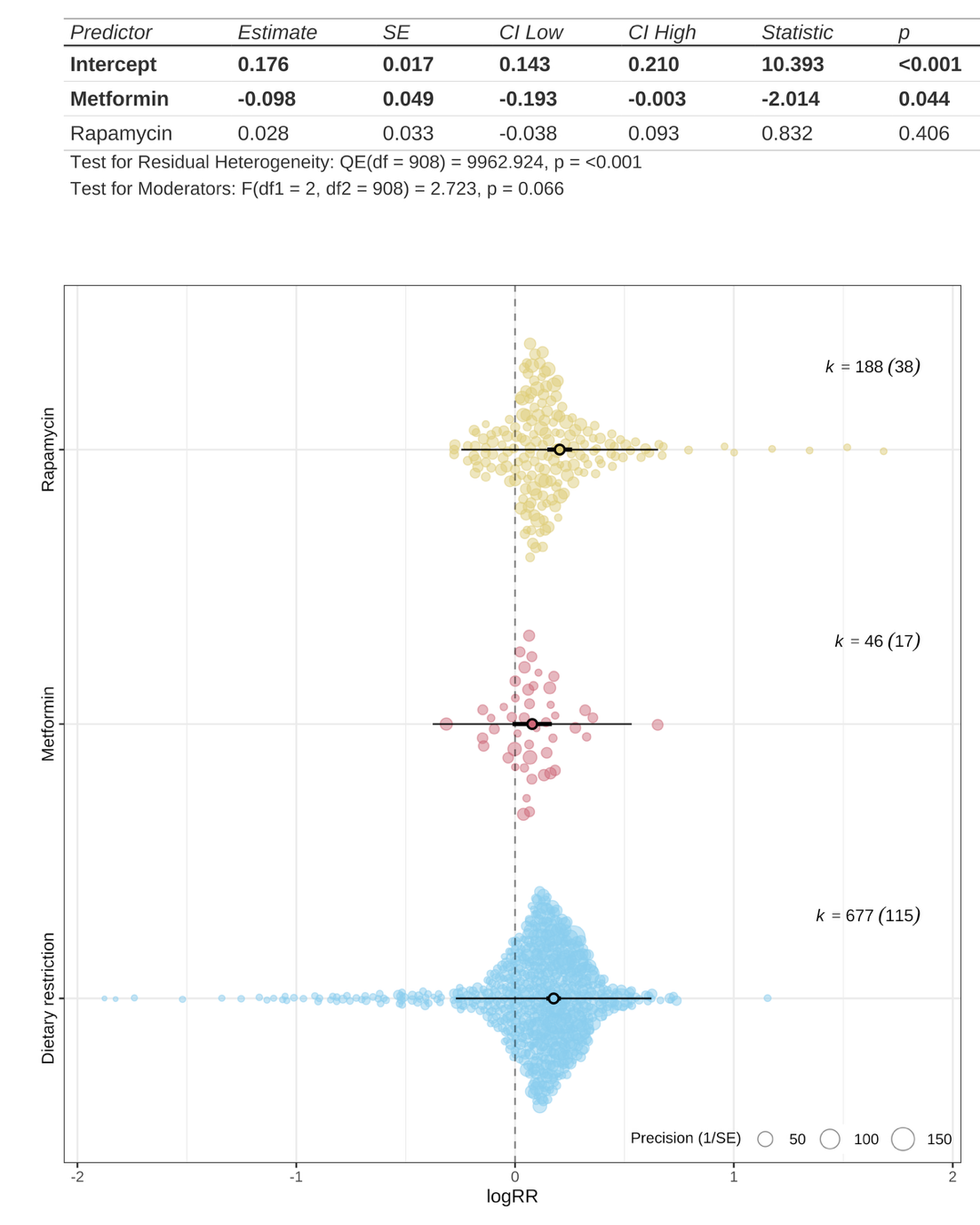

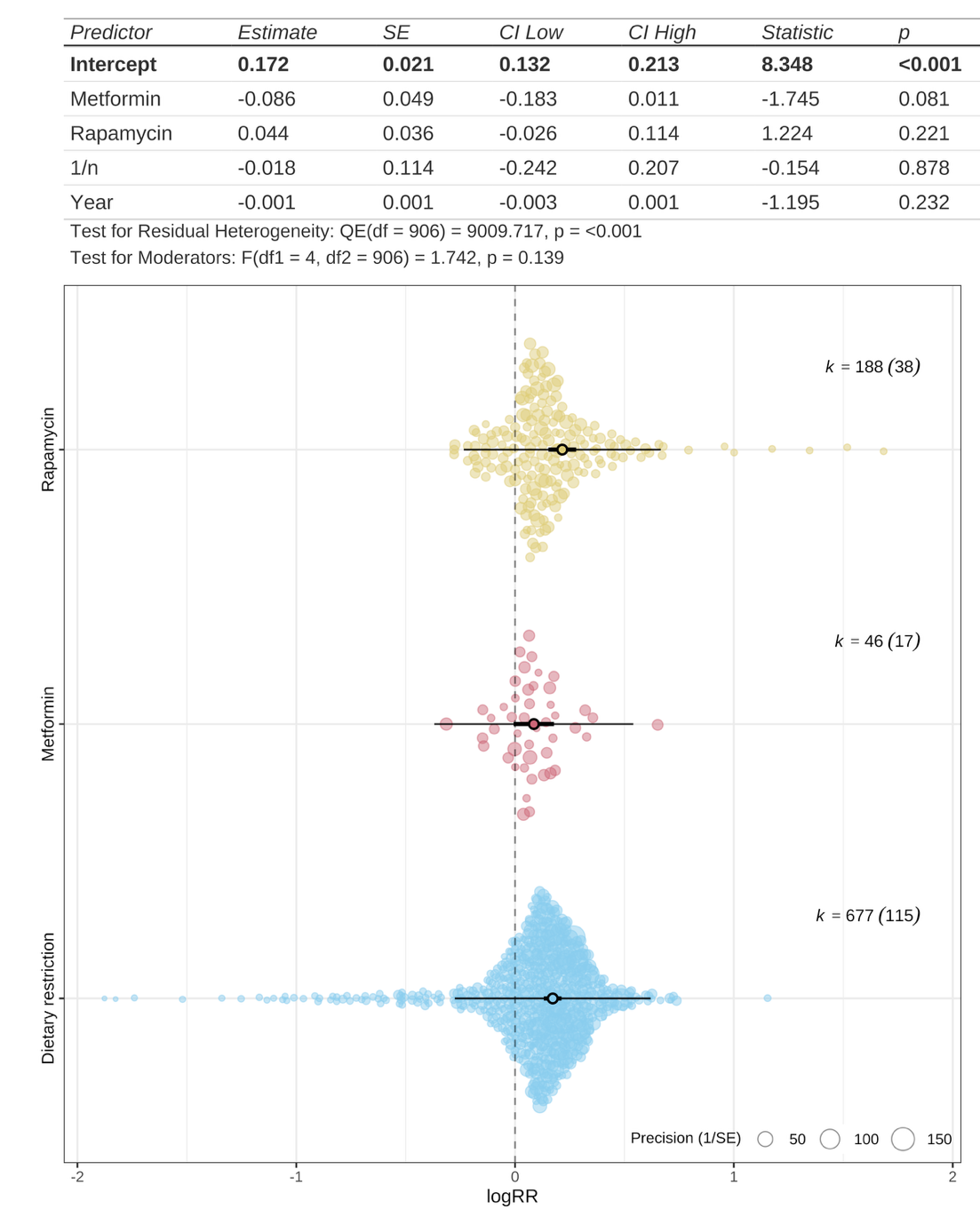
**

**Figure S3.** Model output from a multi-level model of lifespan-extension treatment without (left) and with (right) publication bias correction using both means and medians. Each model is associated with a corresponding model table describing the various predictors, estimates, standard error, low and high 95% confidence intervals, test statistic (*t)*, p value, along with a test for residual heterogeneity and moderators. Bolded rows represent moderators or levels of moderator that are significant (α = 0.050). Below this table is an orchard plot which provides a mean value with surrounding 95% confidence intervals (larger lines) and prediction intervals (thinner lines). The coloured dots represent individual effects sized by precision (1/standard error). The number of effect sizes is given on the right with studies in brackets.


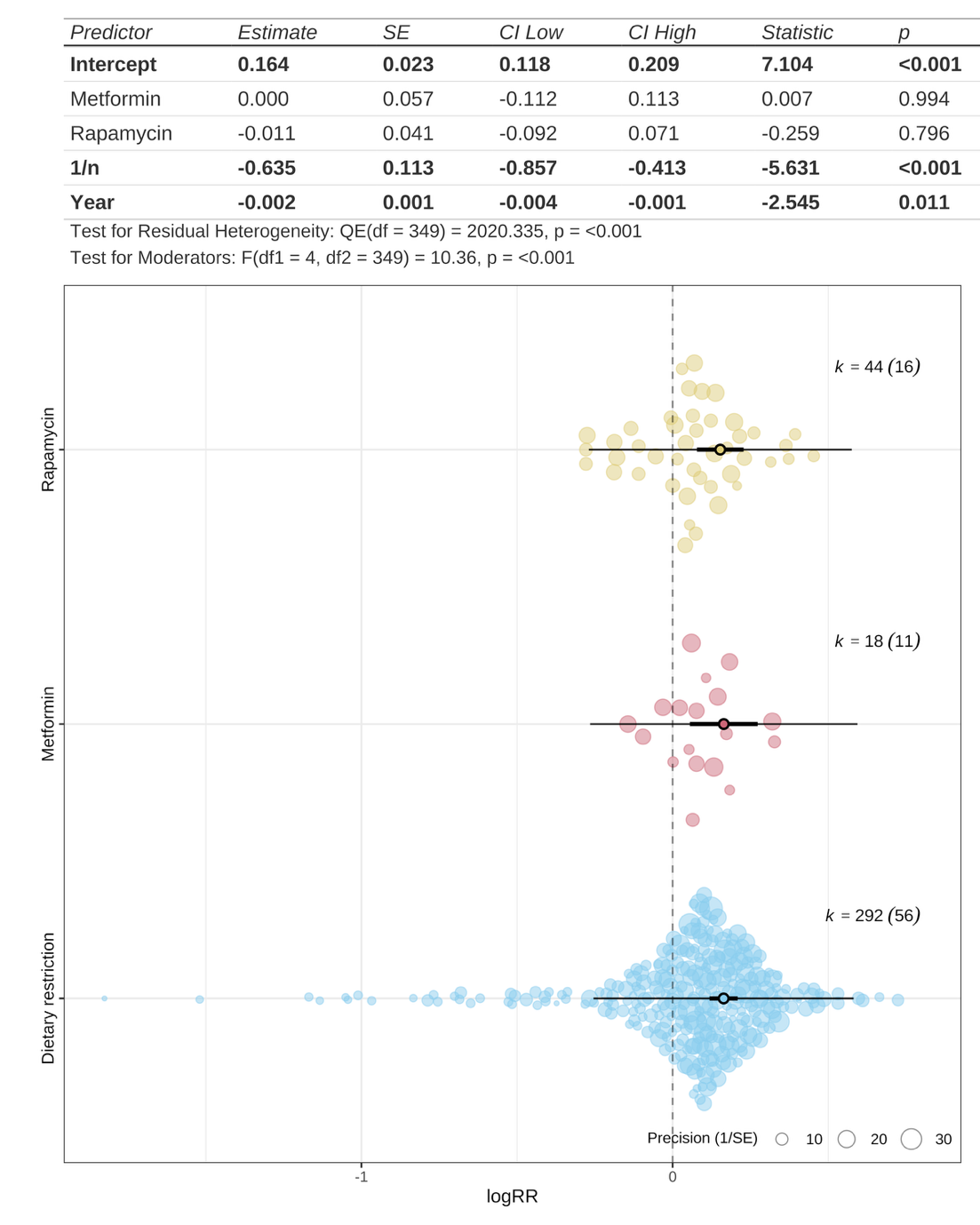

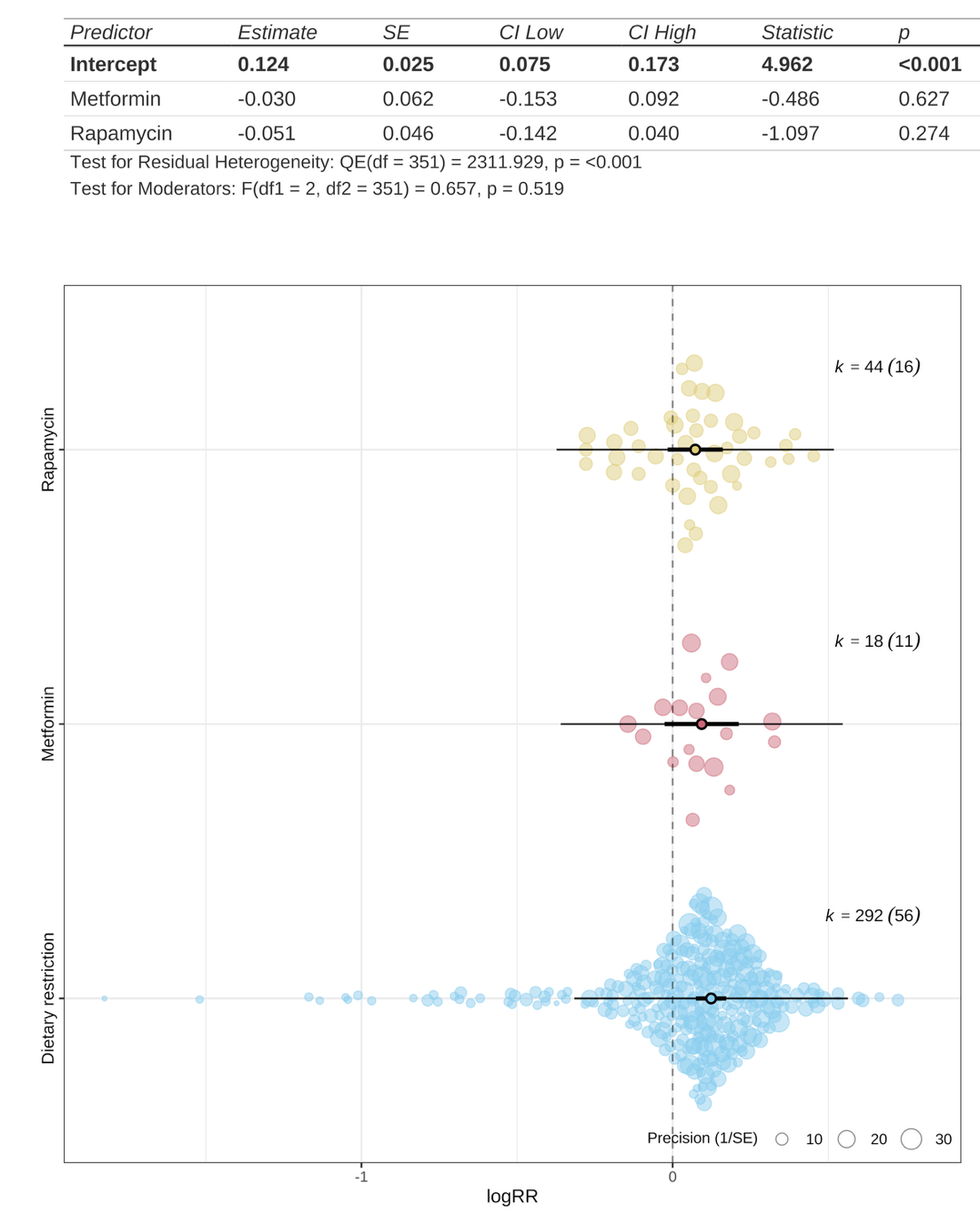


**Figure S4.** Model output from a multi-level model of lifespan-extension treatment without (left) and with (right) publication bias correction using mean values. Each model is associated with a corresponding model table describing the various predictors, estimates, standard error, low and high 95% confidence intervals, test statistic (*t)*, p value, along with a test for residual heterogeneity and moderators. Bolded rows represent moderators or levels of moderator that are significant (α = 0.050). Below this table is an orchard plot which provides a mean value with surrounding 95% confidence intervals (larger lines) and prediction intervals (thinner lines). The coloured dots represent individual effects sized by precision (1/standard error). The number of effect sizes is given on the right with studies in brackets.


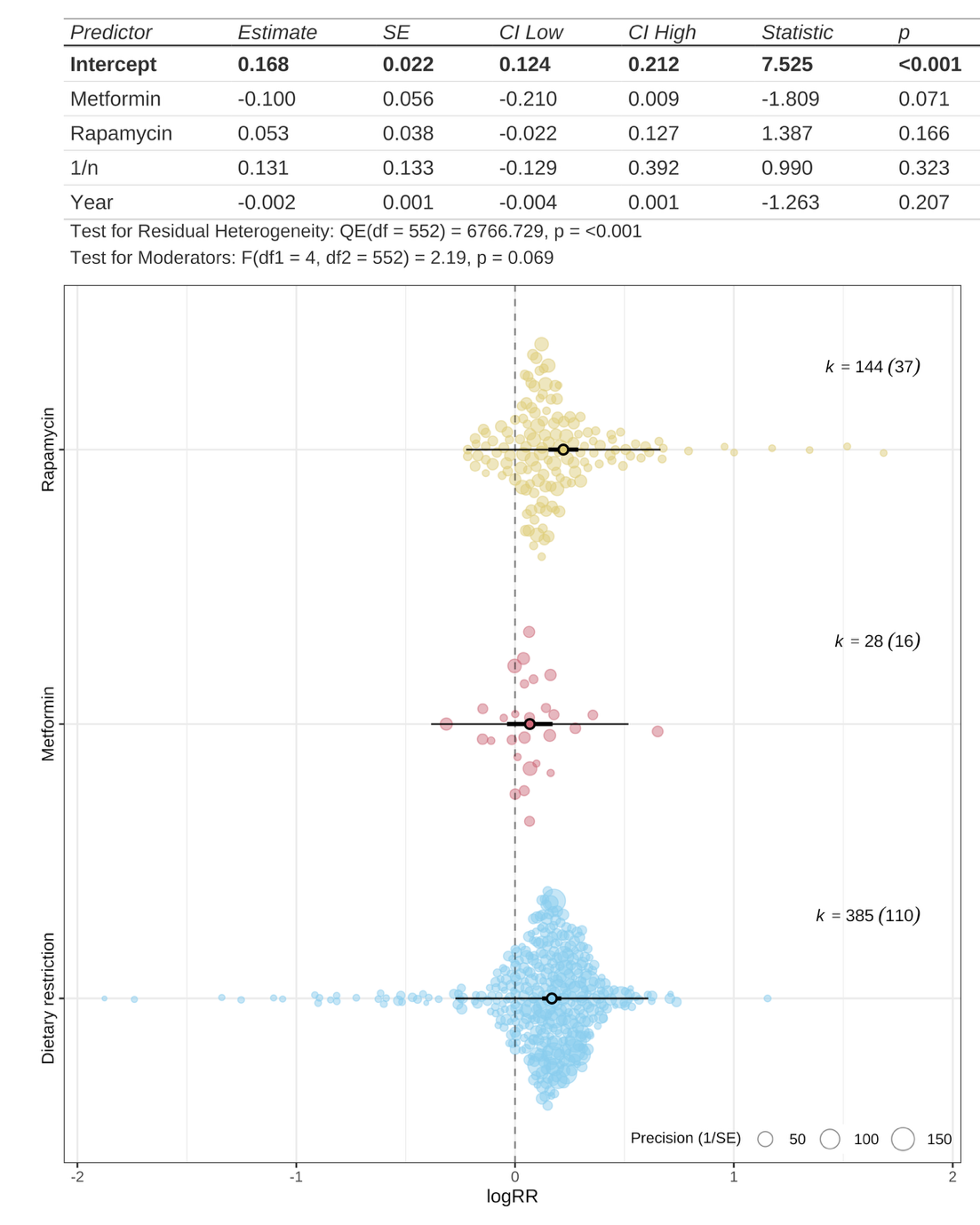

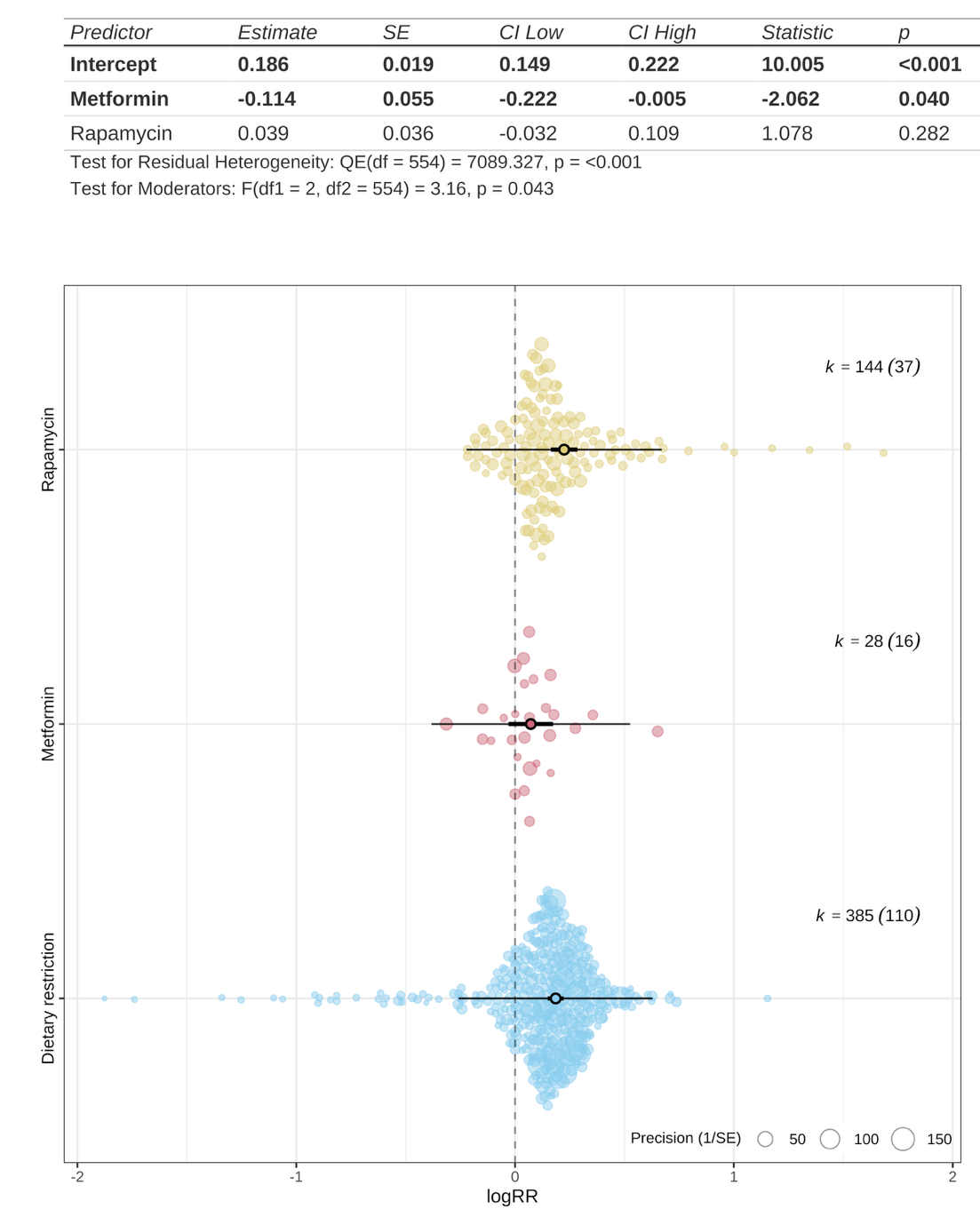


**Figure S5.** Model output from a multi-level model of lifespan-extension treatment without (left) and with (right) publication bias correction using median values. Each model is associated with a corresponding model table describing the various predictors, estimates, standard error, low and high 95% confidence intervals, test statistic (*t)*, p value, along with a test for residual heterogeneity and moderators. Bolded rows represent moderators or levels of moderator that are significant (α = 0.050). Below this table is an orchard plot which provides a mean value with surrounding 95% confidence intervals (larger lines) and prediction intervals (thinner lines). The coloured dots represent individual effects sized by precision (1/standard error). The number of effect sizes is given on the right with studies in brackets.


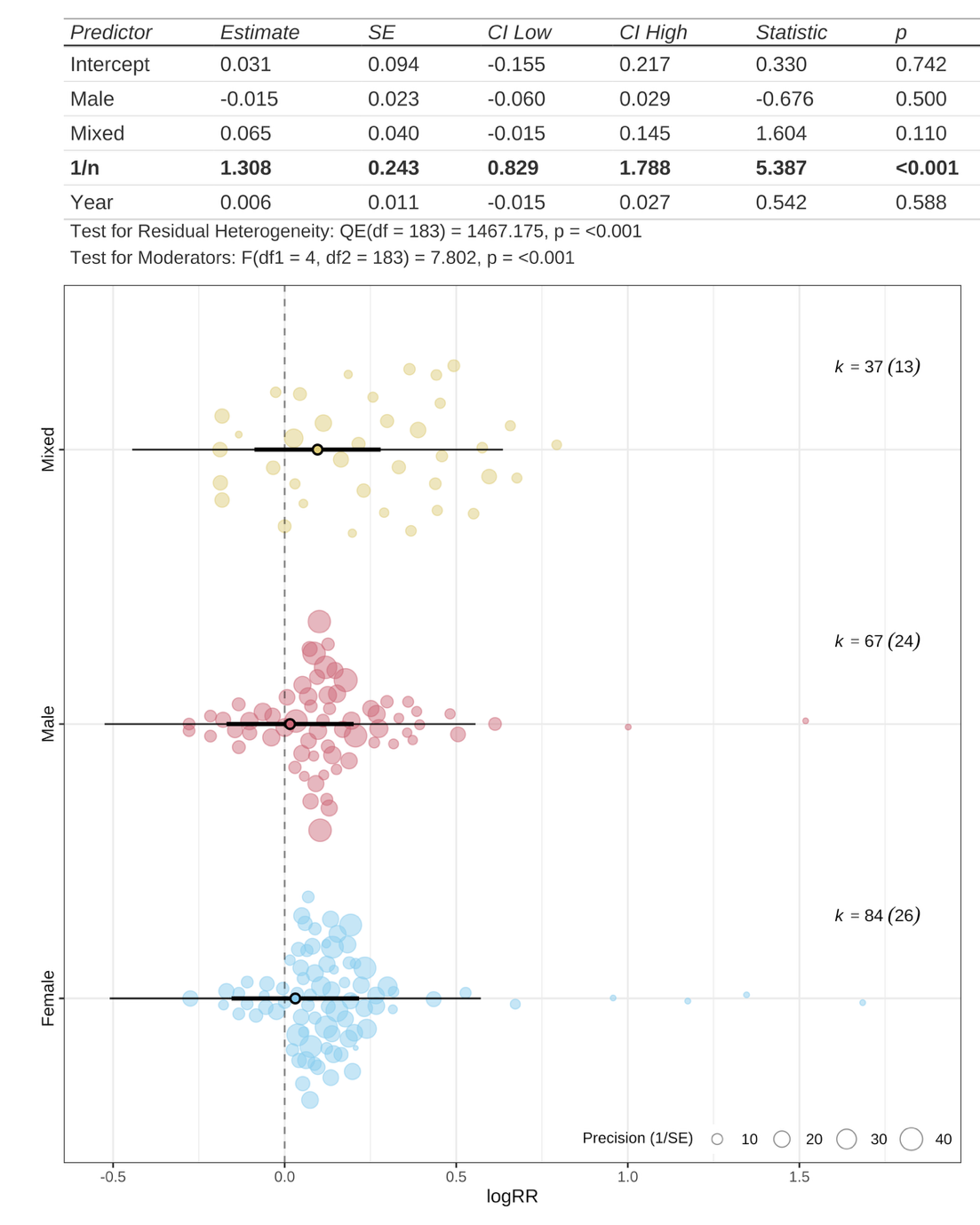

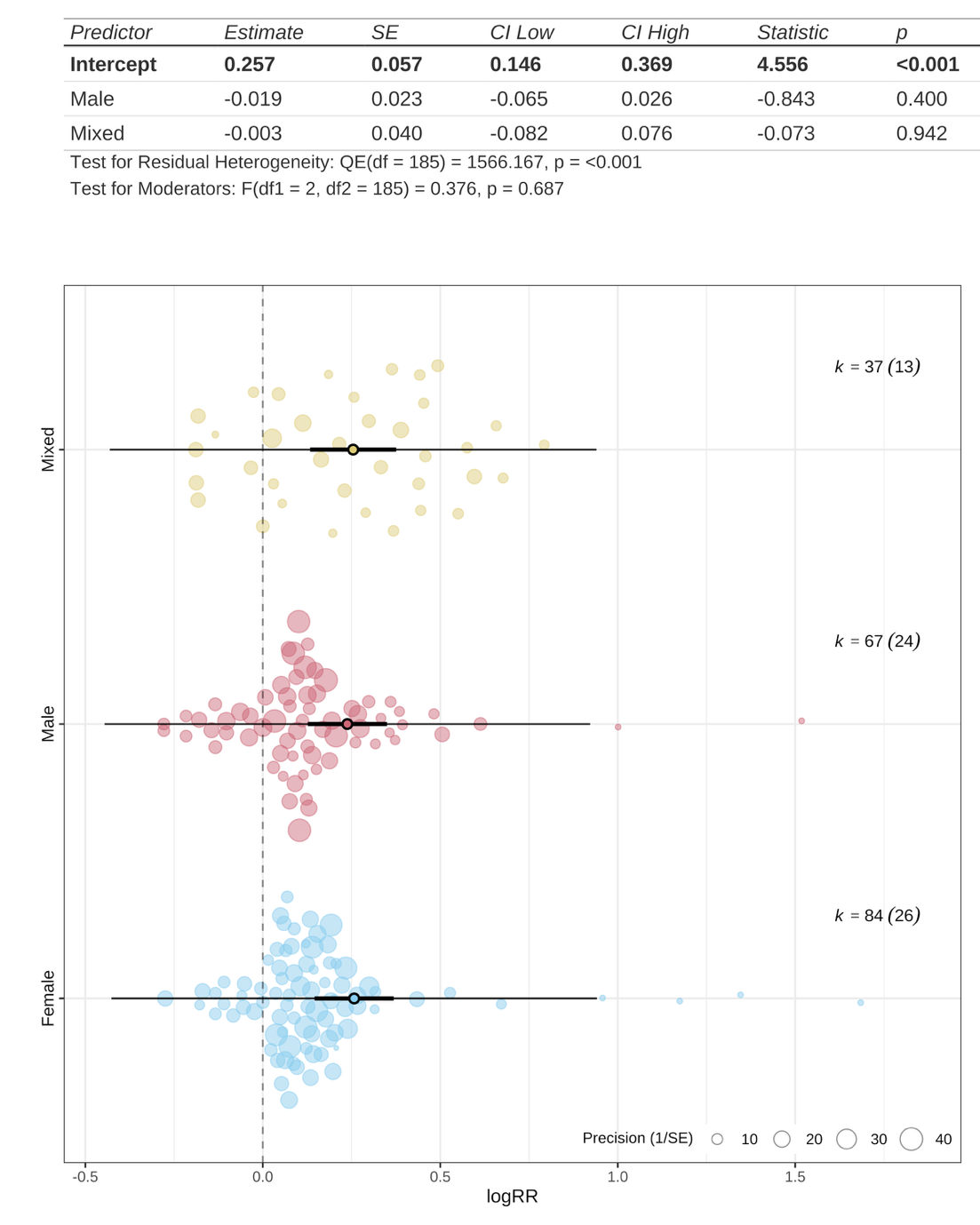


**Figure S6.** Model output from a multi-level model of the effect of sex acting on Rapamycin without (left) and with (right) publication bias correction using both mean and median values. Each model is associated with a corresponding model table describing the various predictors, estimates, standard error, low and high 95% confidence intervals, test statistic (*t)*, p value, along with a test for residual heterogeneity and moderators. Bolded rows represent moderators or levels of moderator that are significant (α = 0.050). Below this table is an orchard plot which provides a mean value with surrounding 95% confidence intervals (larger lines) and prediction intervals (thinner lines). The coloured dots represent individual effects sized by precision (1/standard error). The number of effect sizes is given on the right with studies in brackets.


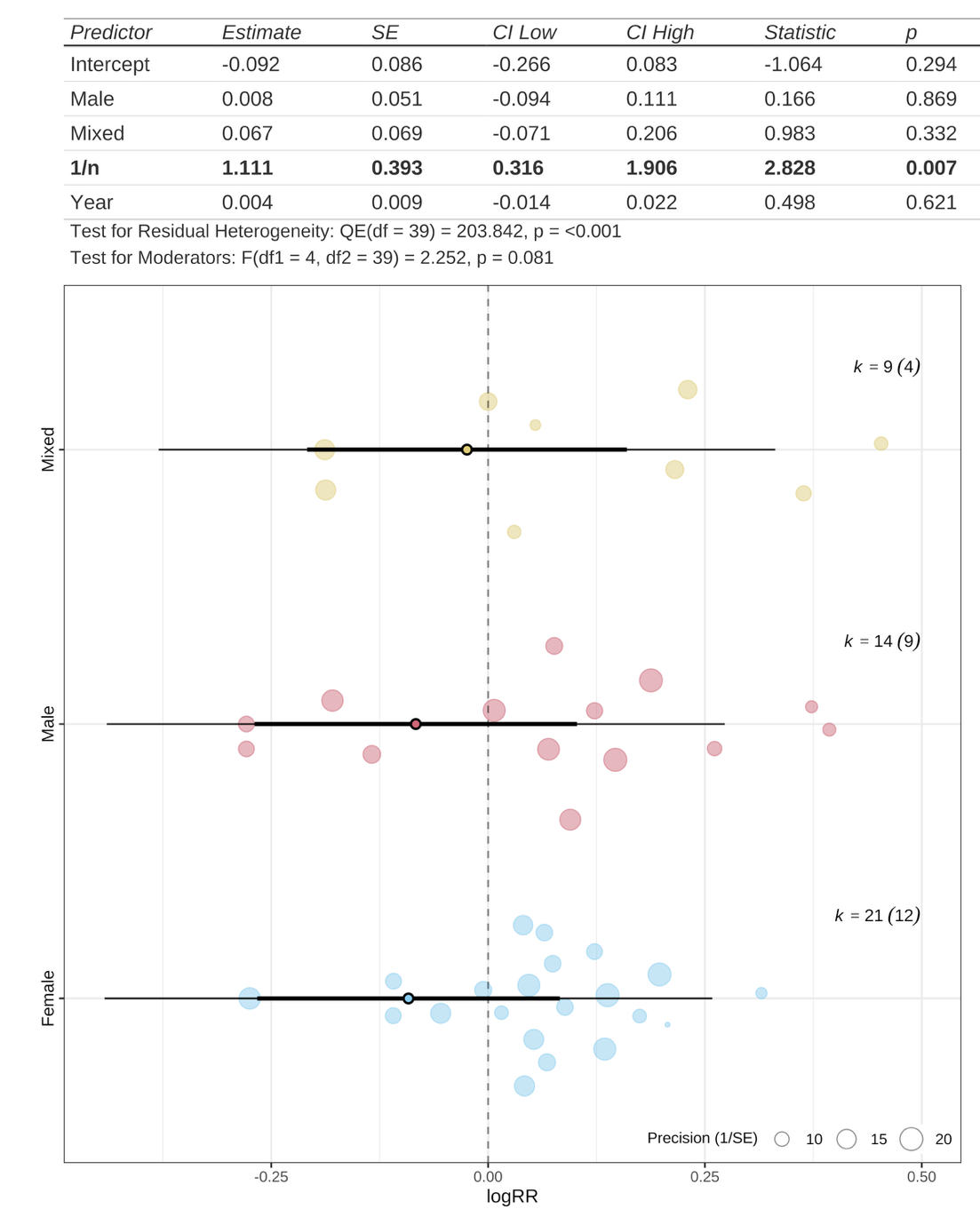

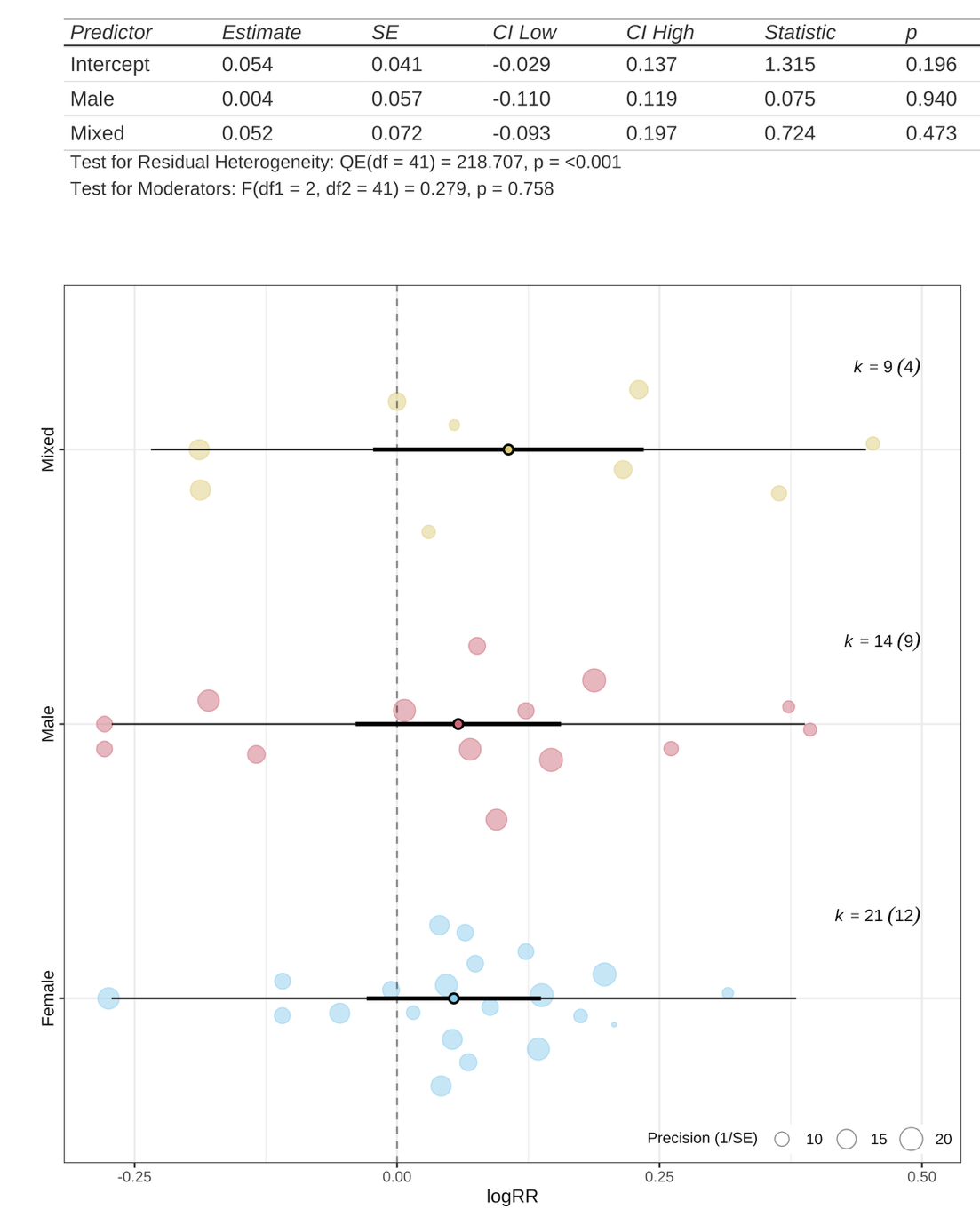


**Figure S7.** Model output from a multi-level model of the effect of sex acting on Rapamycin without (left) and with (right) publication bias correction using mean values. Each model is associated with a corresponding model table describing the various predictors, estimates, standard error, low and high 95% confidence intervals, test statistic (*t)*, p value, along with a test for residual heterogeneity and moderators. Bolded rows represent moderators or levels of moderator that are significant (α = 0.050). Below this table is an orchard plot which provides a mean value with surrounding 95% confidence intervals (larger lines) and prediction intervals (thinner lines). The coloured dots represent individual effects sized by precision (1/standard error). The number of effect sizes is given on the right with studies in brackets.


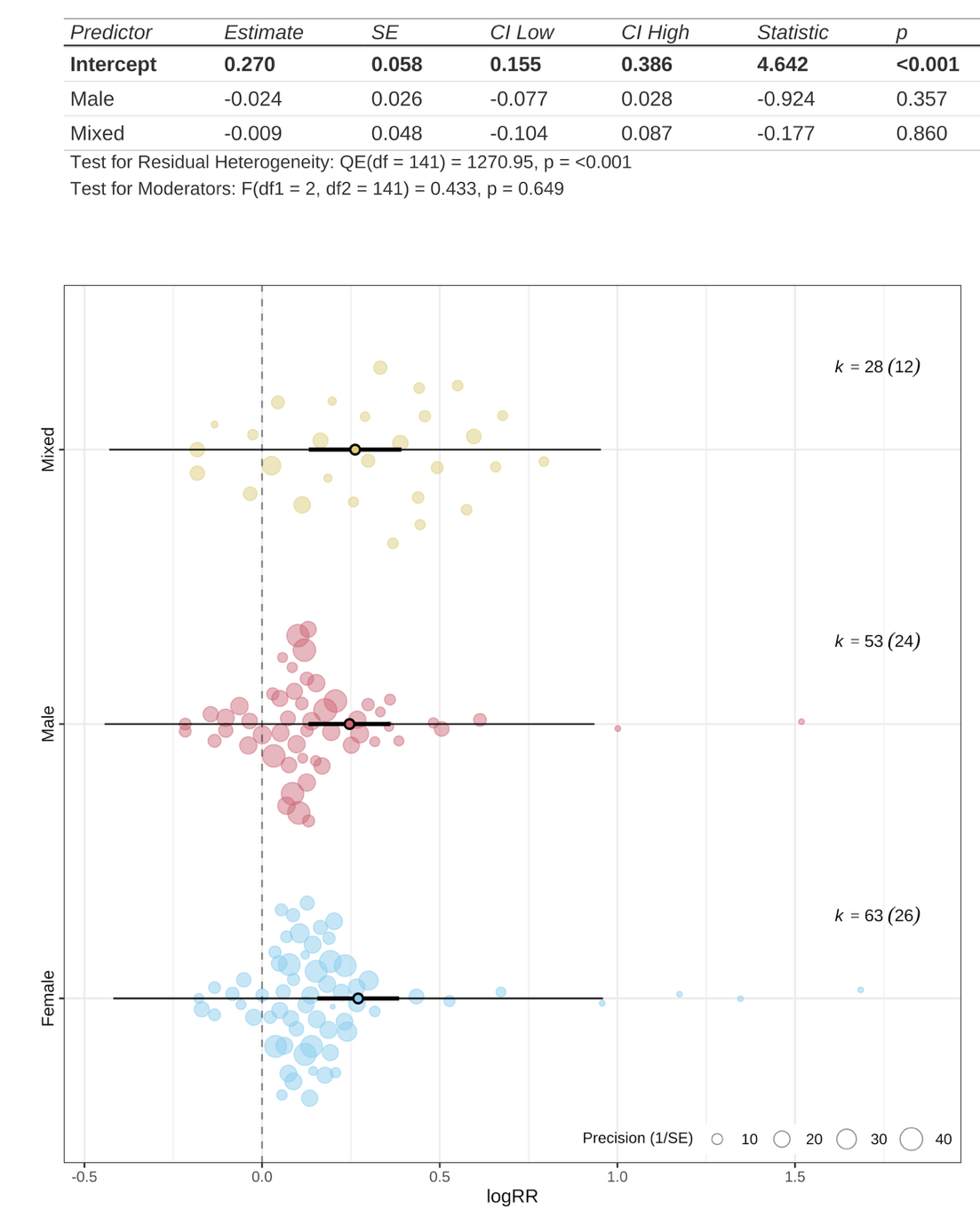

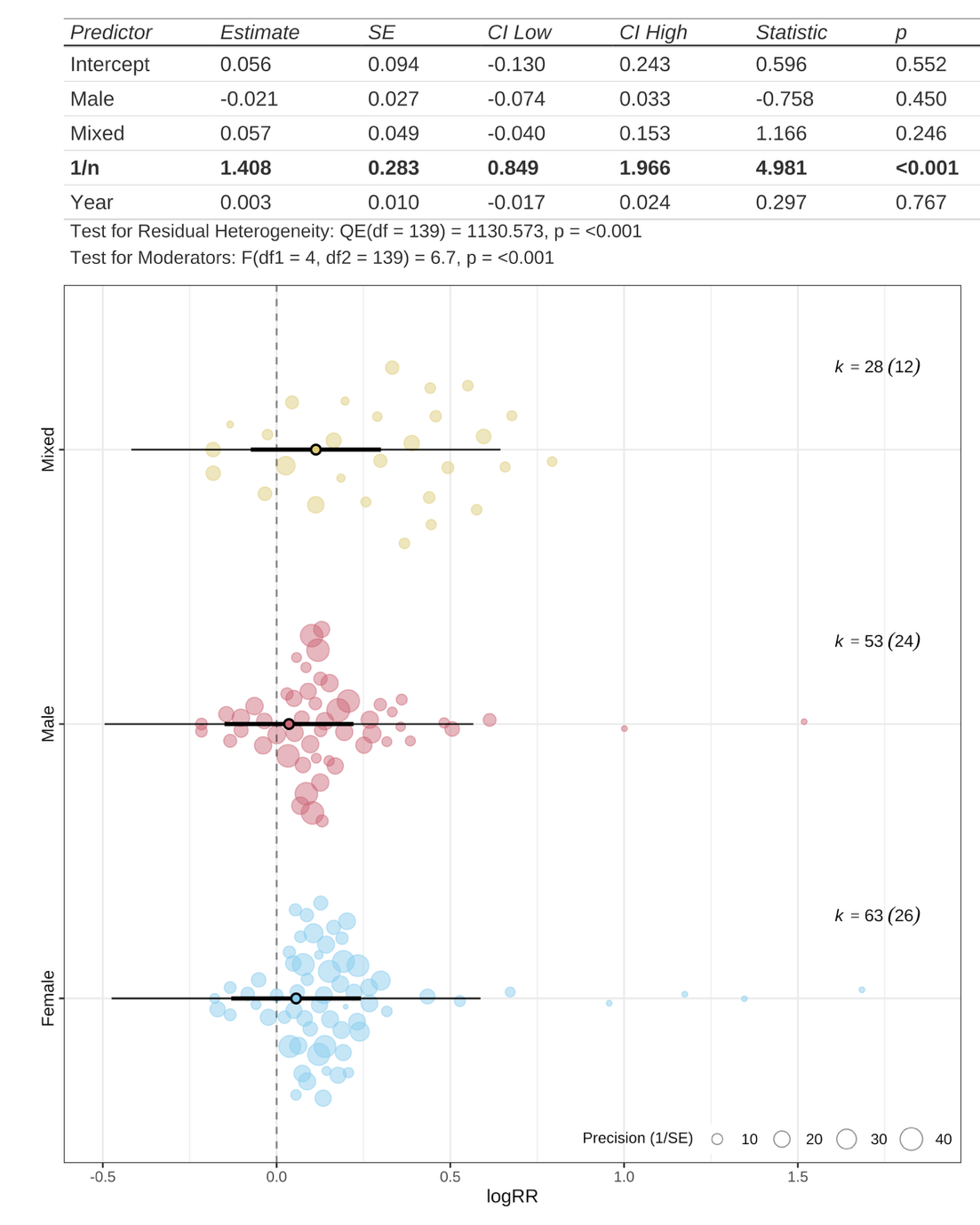


**Figure S8.** Model output from a multi-level model of the effect of sex acting on Rapamycin without (left) and with (right) publication bias correction using median values. Each model is associated with a corresponding model table describing the various predictors, estimates, standard error, low and high 95% confidence intervals, test statistic (*t)*, p value, along with a test for residual heterogeneity and moderators. Bolded rows represent moderators or levels of moderator that are significant (α = 0.050). Below this table is an orchard plot which provides a mean value with surrounding 95% confidence intervals (larger lines) and prediction intervals (thinner lines). The coloured dots represent individual effects sized by precision (1/standard error). The number of effect sizes is given on the right with studies in brackets.


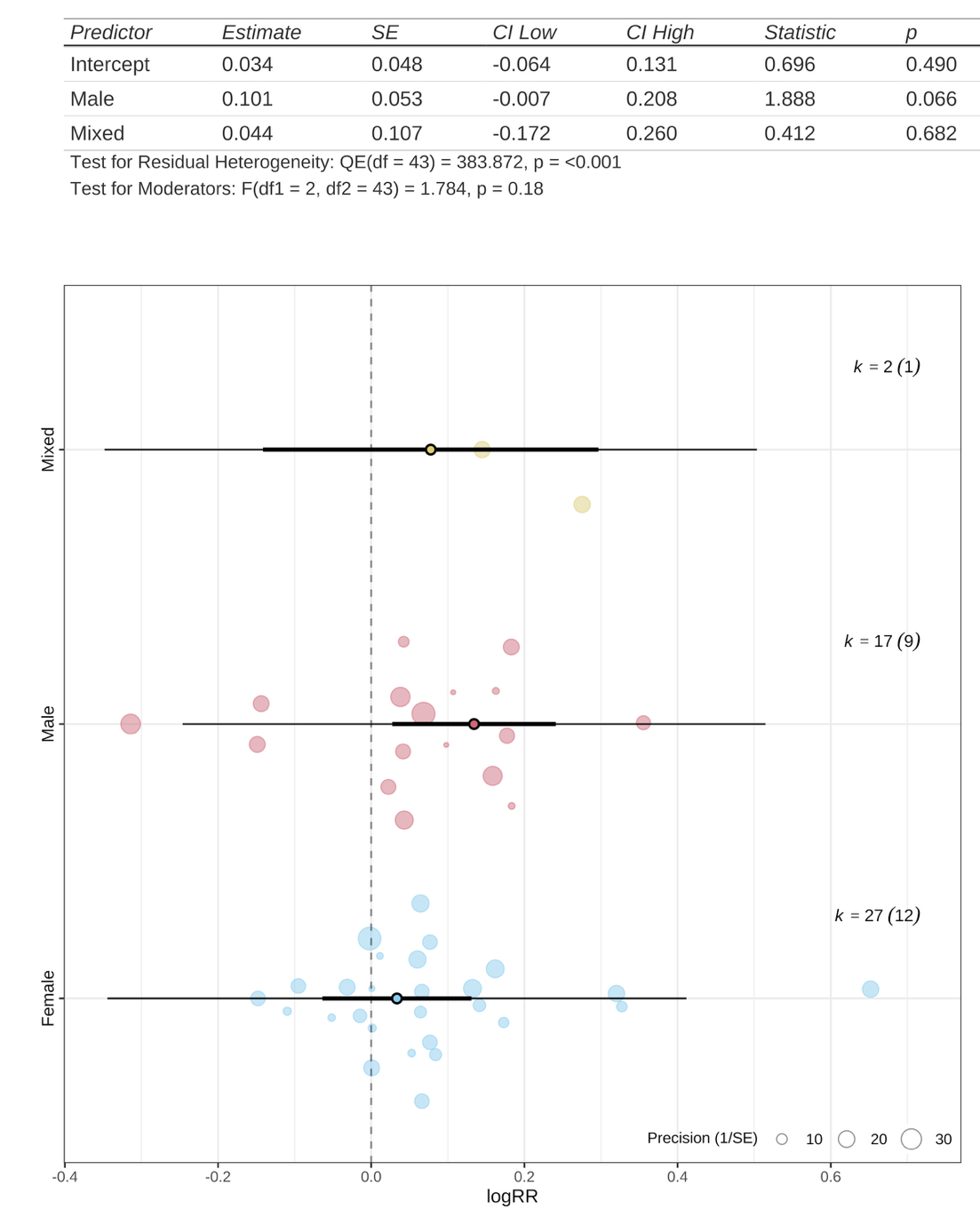

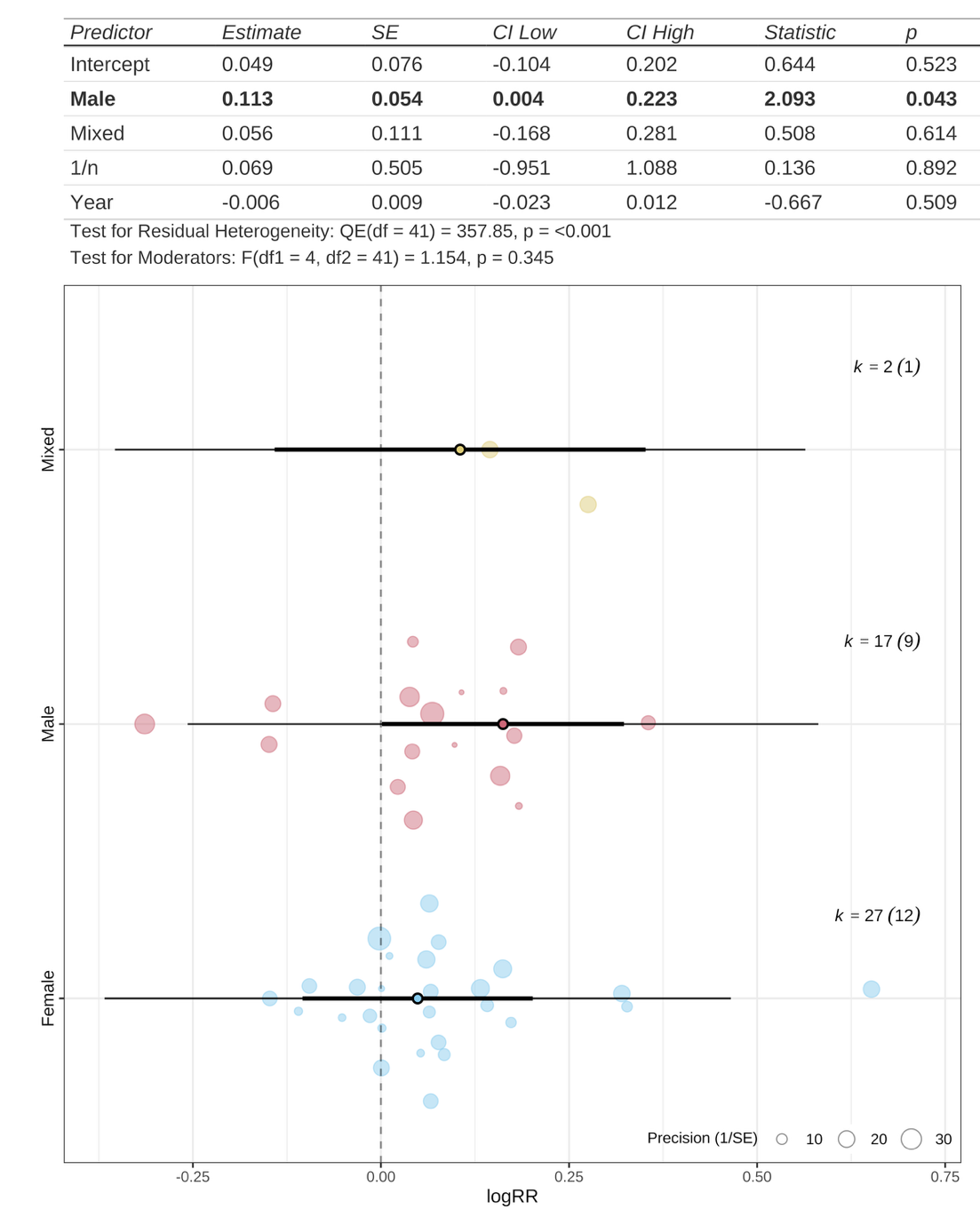


**Figure S9.** Model output from a multi-level model of the effect of sex acting on Metformin without (left) and with (right) publication bias correction using both mean and median values. Each model is associated with a corresponding model table describing the various predictors, estimates, standard error, low and high 95% confidence intervals, test statistic (*t)*, p value, along with a test for residual heterogeneity and moderators. Bolded rows represent moderators or levels of moderator that are significant (α = 0.050). Below this table is an orchard plot which provides a mean value with surrounding 95% confidence intervals (larger lines) and prediction intervals (thinner lines). The coloured dots represent individual effects sized by precision (1/standard error). The number of effect sizes is given on the right with studies in brackets.


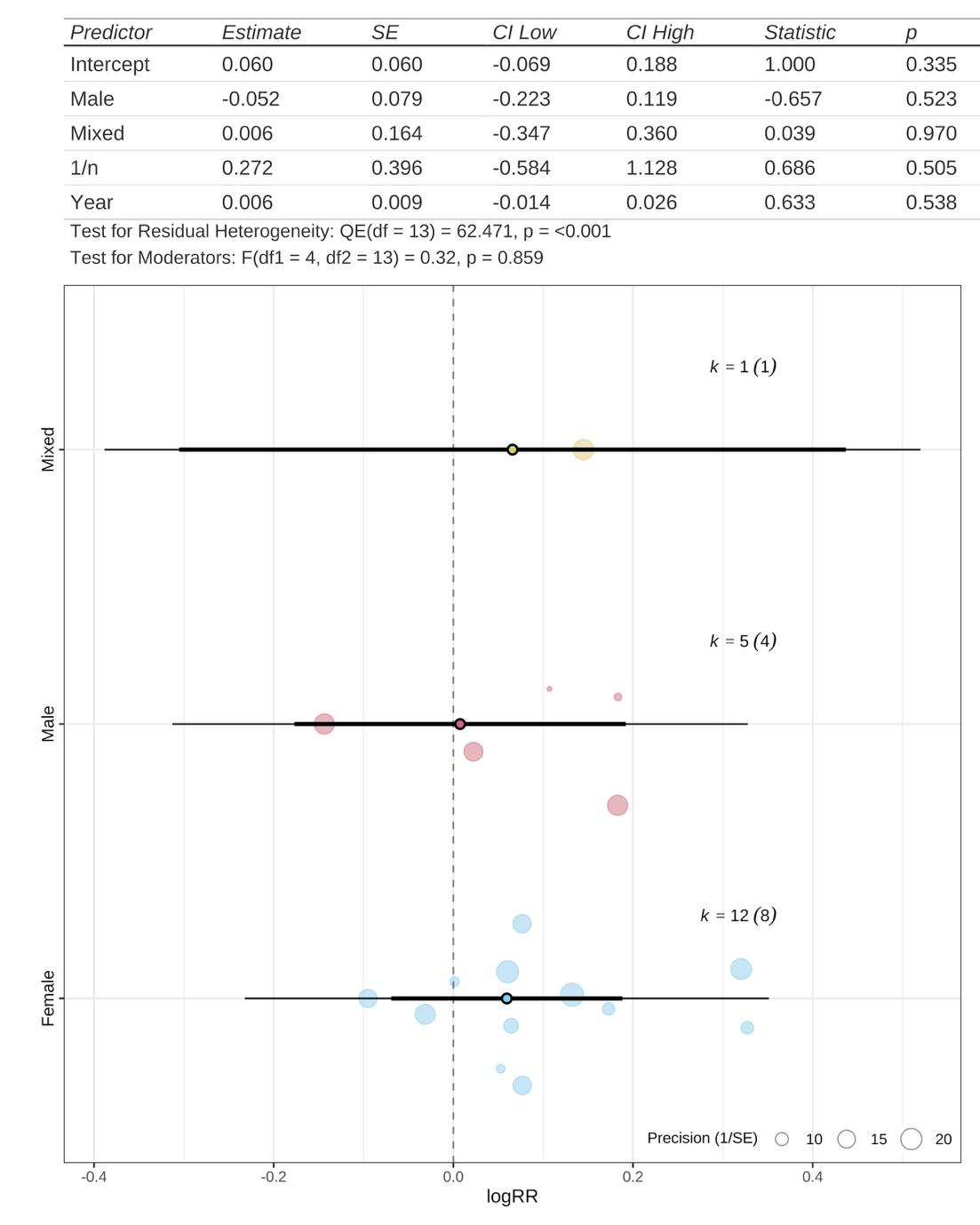

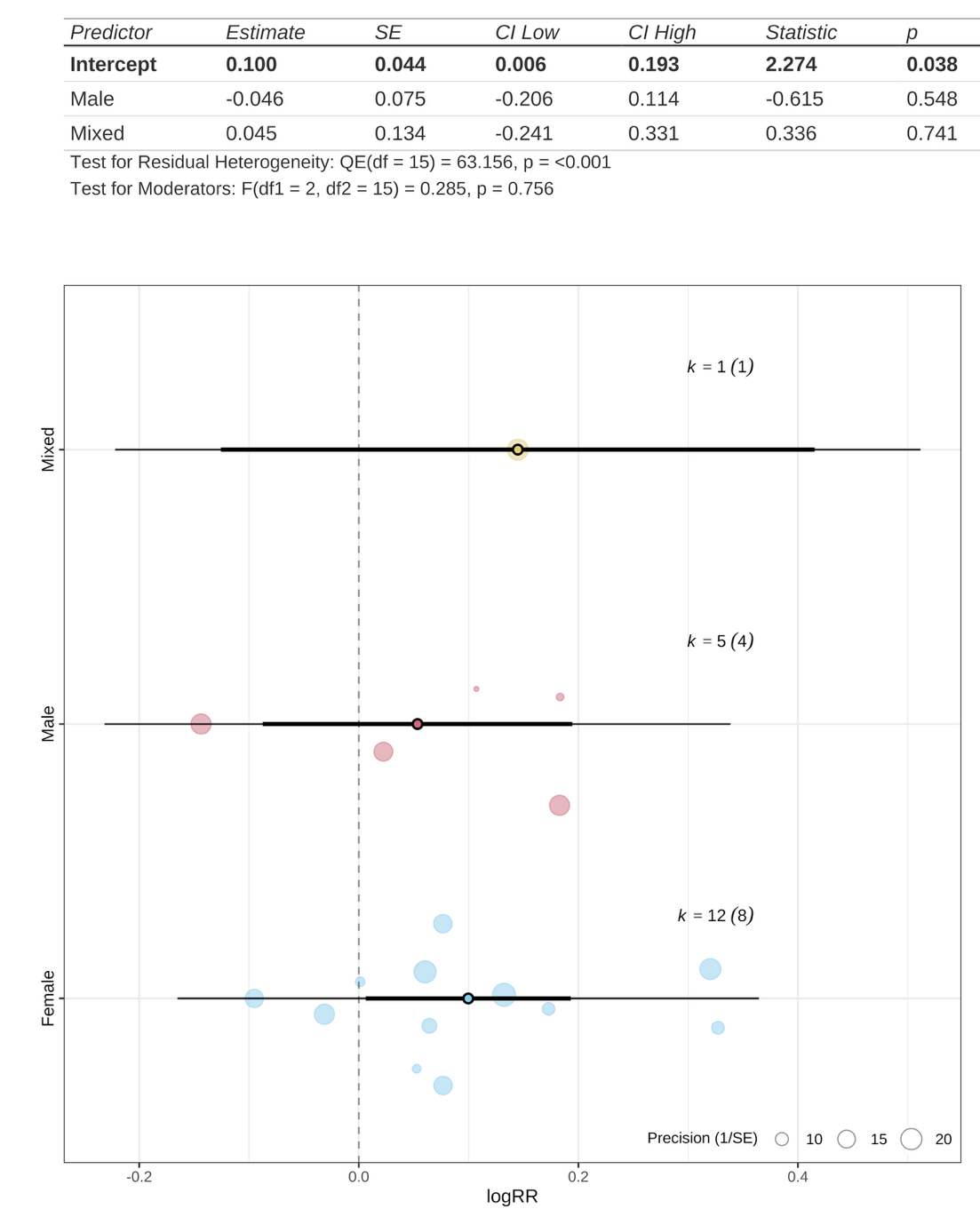


**Figure S10.** Model output from a multi-level model of the effect of sex acting on Metformin without (left) and with (right) publication bias correction using mean values. Each model is associated with a corresponding model table describing the various predictors, estimates, standard error, low and high 95% confidence intervals, test statistic (*t)*, p value, along with a test for residual heterogeneity and moderators. Bolded rows represent moderators or levels of moderator that are significant (α = 0.050). Below this table is an orchard plot which provides a mean value with surrounding 95% confidence intervals (larger lines) and prediction intervals (thinner lines). The coloured dots represent individual effects sized by precision (1/standard error). The number of effect sizes is given on the right with studies in brackets.


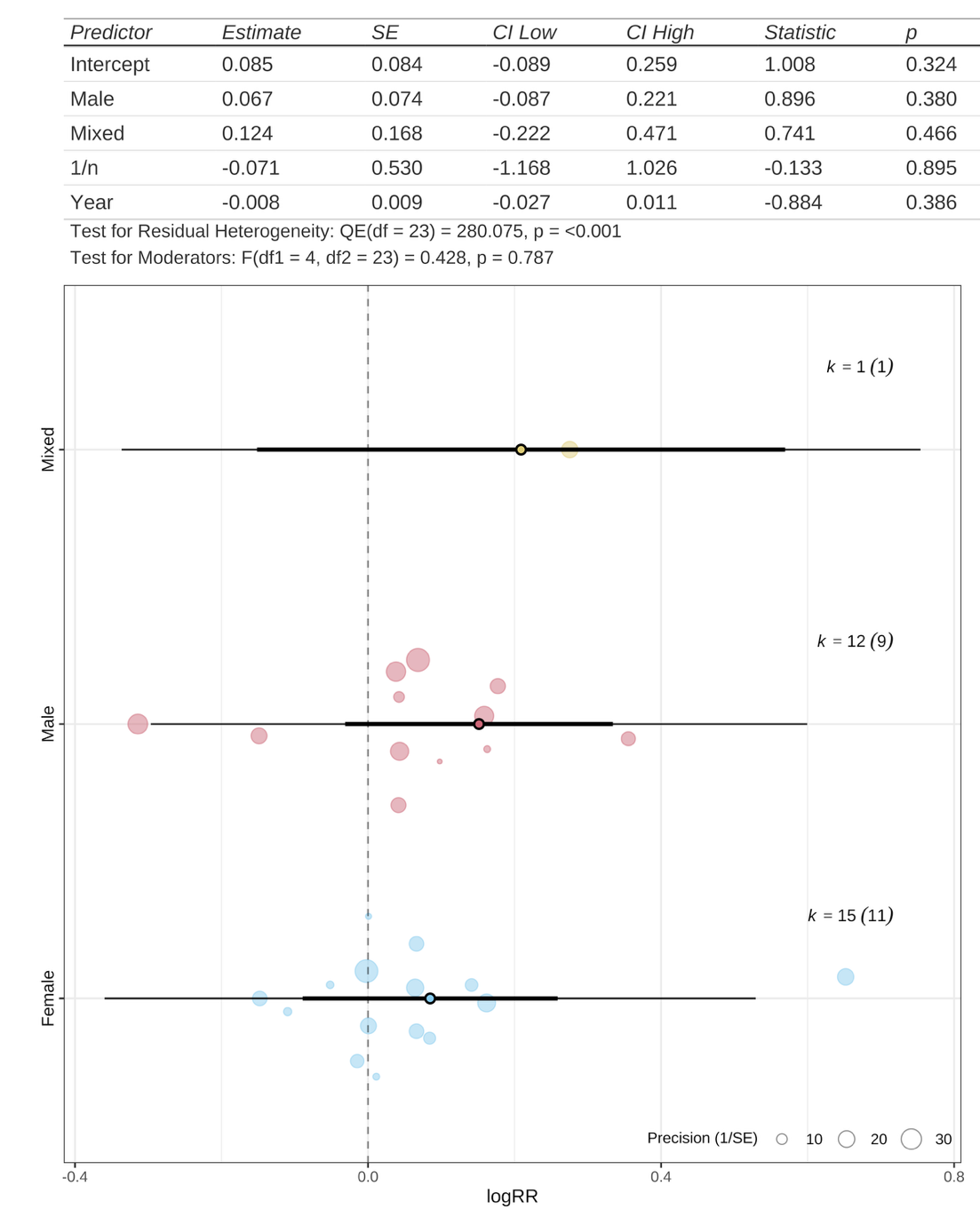

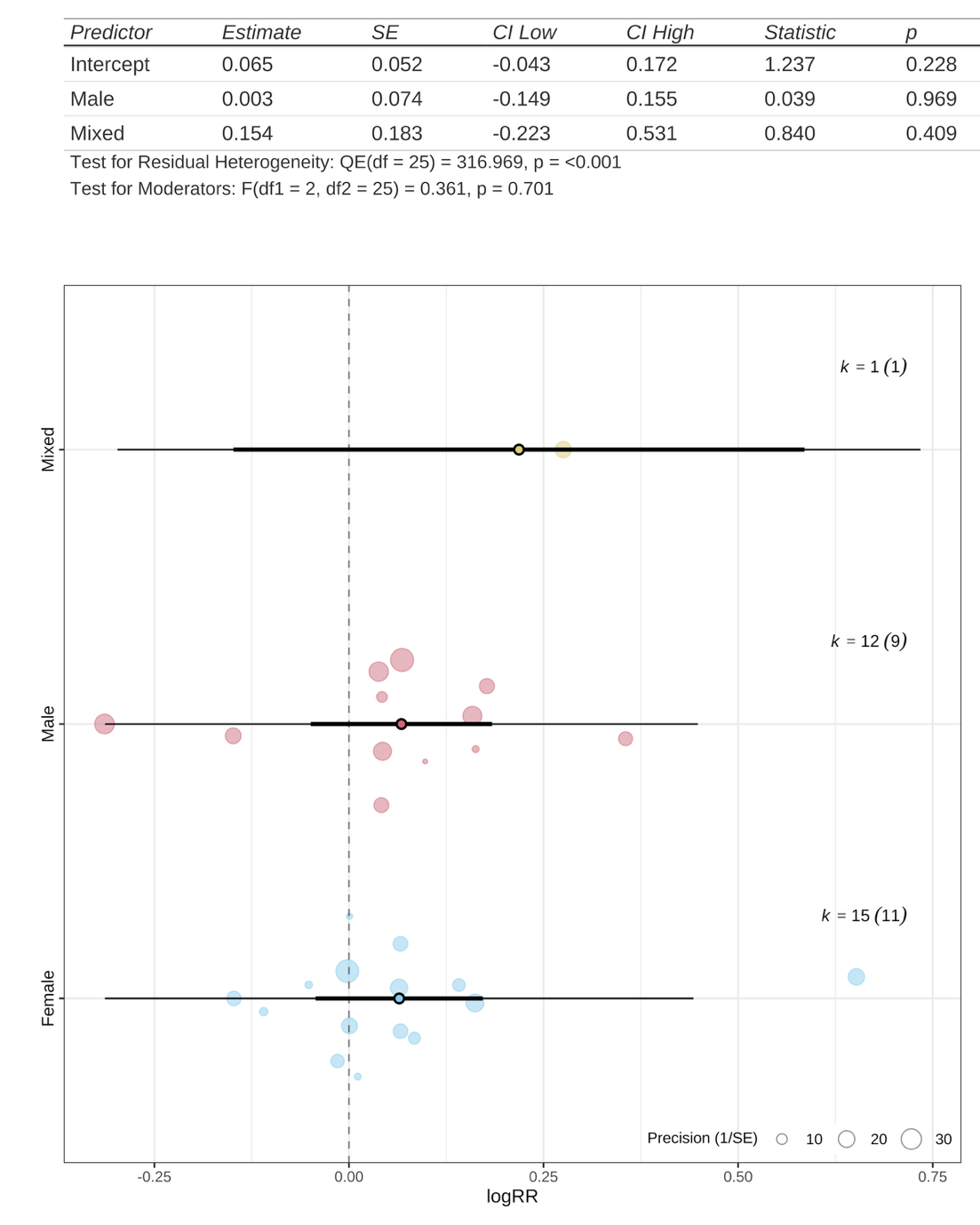


**Figure S11.** Model output from a multi-level model of the effect of sex acting on Metformin without (left) and with (right) publication bias correction using median values. Each model is associated with a corresponding model table describing the various predictors, estimates, standard error, low and high 95% confidence intervals, test statistic (*t)*, p value, along with a test for residual heterogeneity and moderators. Bolded rows represent moderators or levels of moderator that are significant (α = 0.050). Below this table is an orchard plot which provides a mean value with surrounding 95% confidence intervals (larger lines) and prediction intervals (thinner lines). The coloured dots represent individual effects sized by precision (1/standard error). The number of effect sizes is given on the right with studies in brackets.


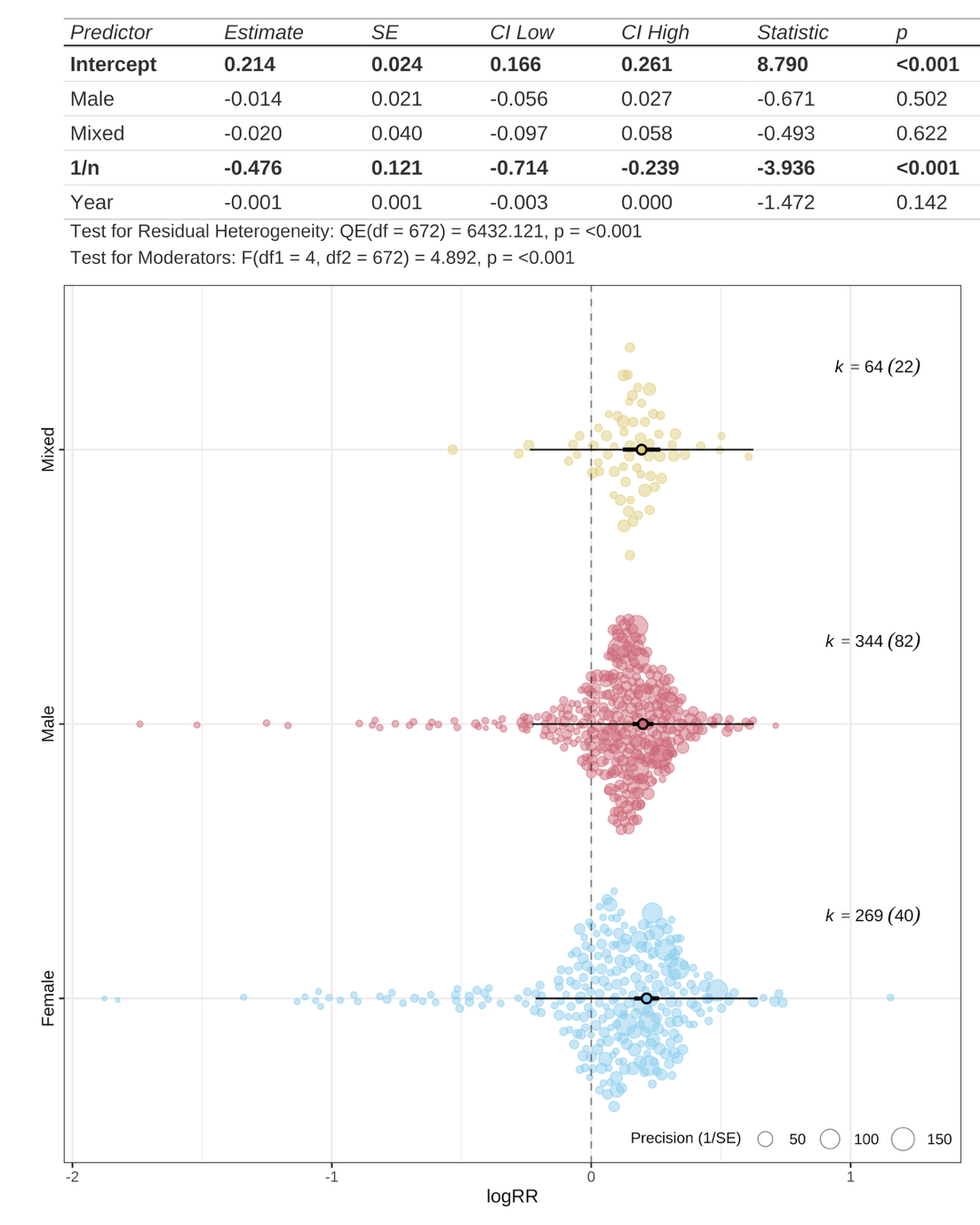

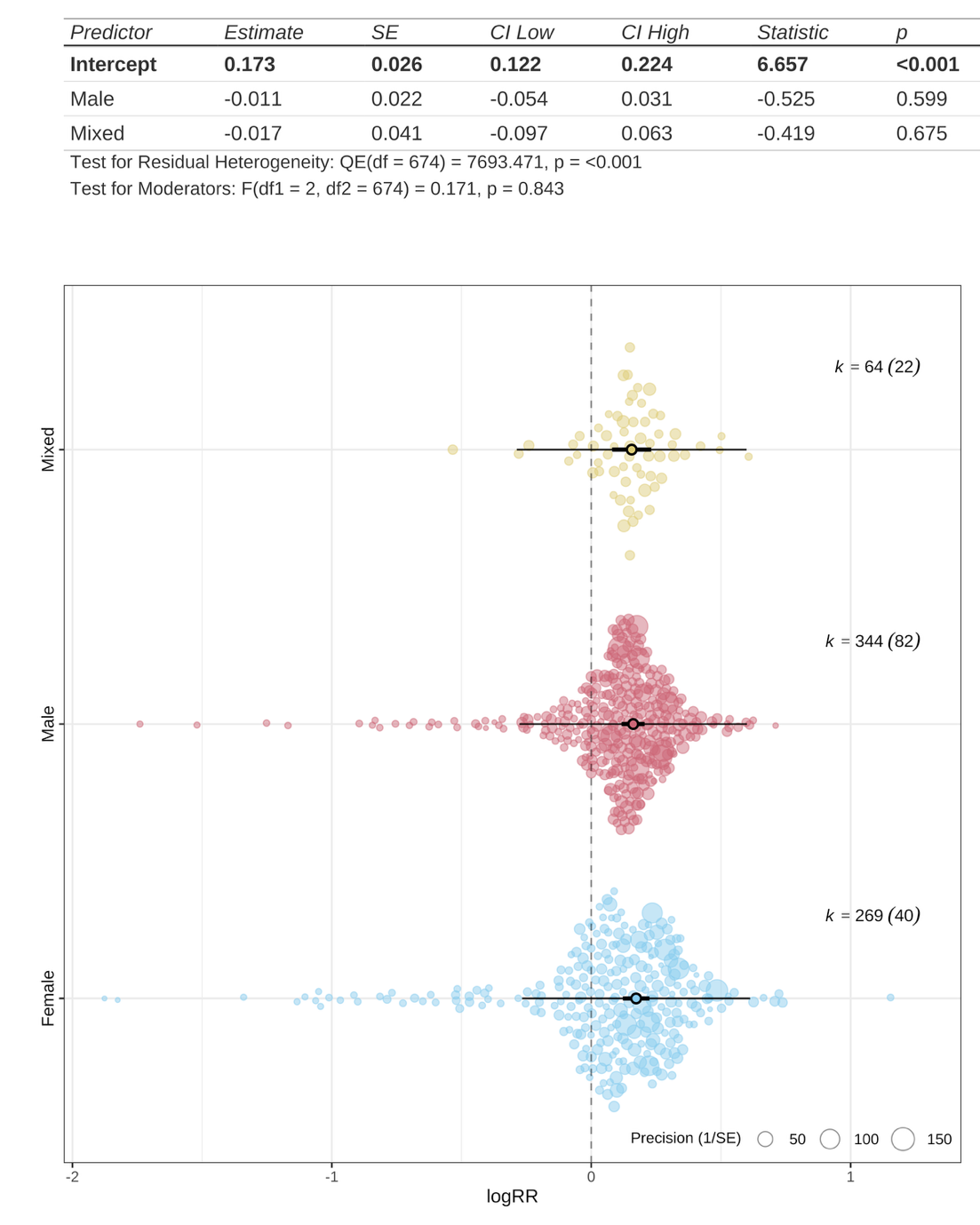


**Figure S12.** Model output from a multi-level model of the effect of sex acting on dietary restriction without (left) and with (right) publication bias correction using both mean and median values. Each model is associated with a corresponding model table describing the various predictors, estimates, standard error, low and high 95% confidence intervals, test statistic (*t)*, p value, along with a test for residual heterogeneity and moderators. Bolded rows represent moderators or levels of moderator that are significant (α = 0.050). Below this table is an orchard plot which provides a mean value with surrounding 95% confidence intervals (larger lines) and prediction intervals (thinner lines). The coloured dots represent individual effects sized by precision (1/standard error). The number of effect sizes is given on the right with studies in brackets.


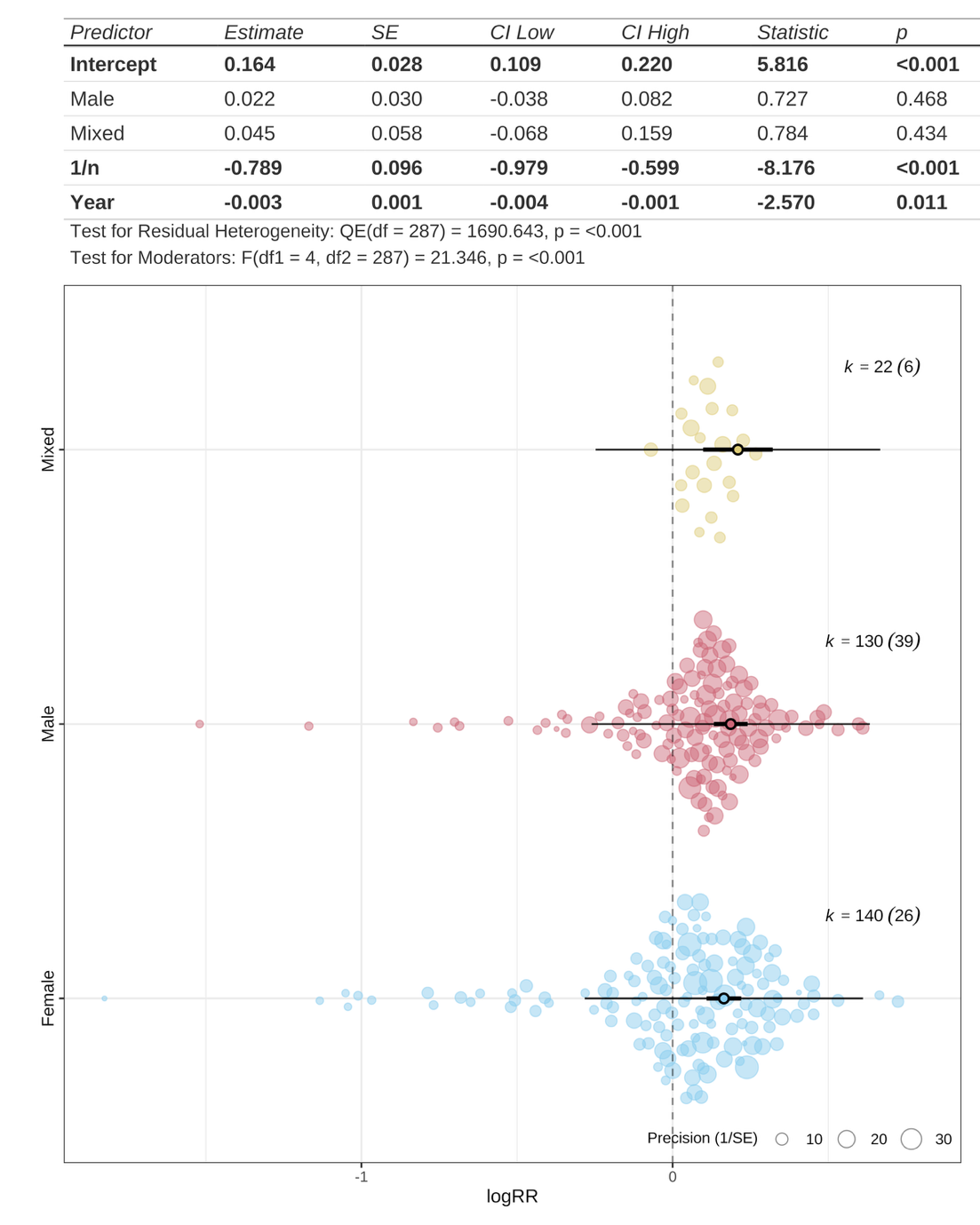

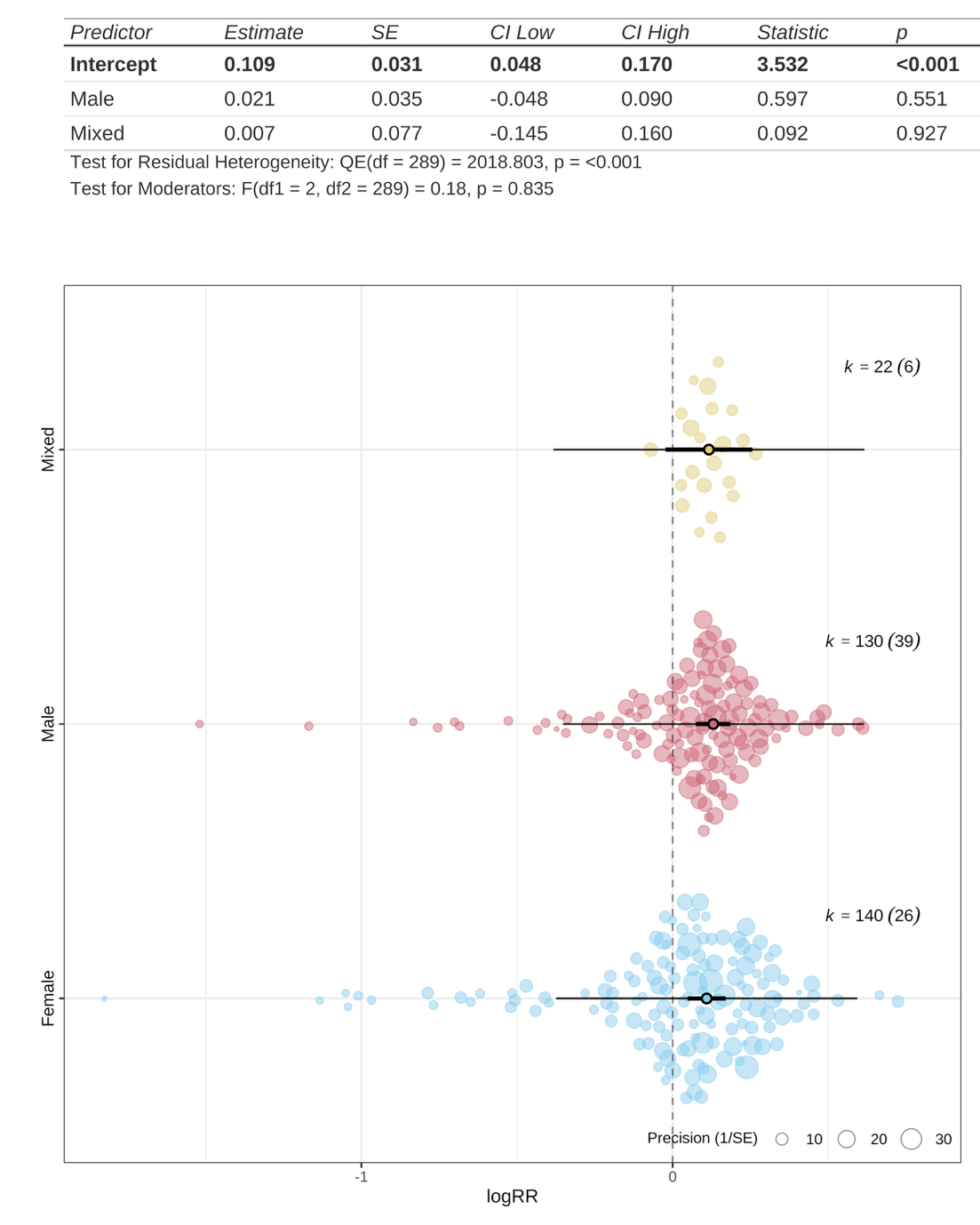


**Figure S13.** Model output from a multi-level model of the effect of sex acting on dietary restriction without (left) and with (right) publication bias correction using mean values. Each model is associated with a corresponding model table describing the various predictors, estimates, standard error, low and high 95% confidence intervals, test statistic (*t)*, p value, along with a test for residual heterogeneity and moderators. Bolded rows represent moderators or levels of moderator that are significant (α = 0.050). Below this table is an orchard plot which provides a mean value with surrounding 95% confidence intervals (larger lines) and prediction intervals (thinner lines). The coloured dots represent individual effects sized by precision (1/standard error). The number of effect sizes is given on the right with studies in brackets.


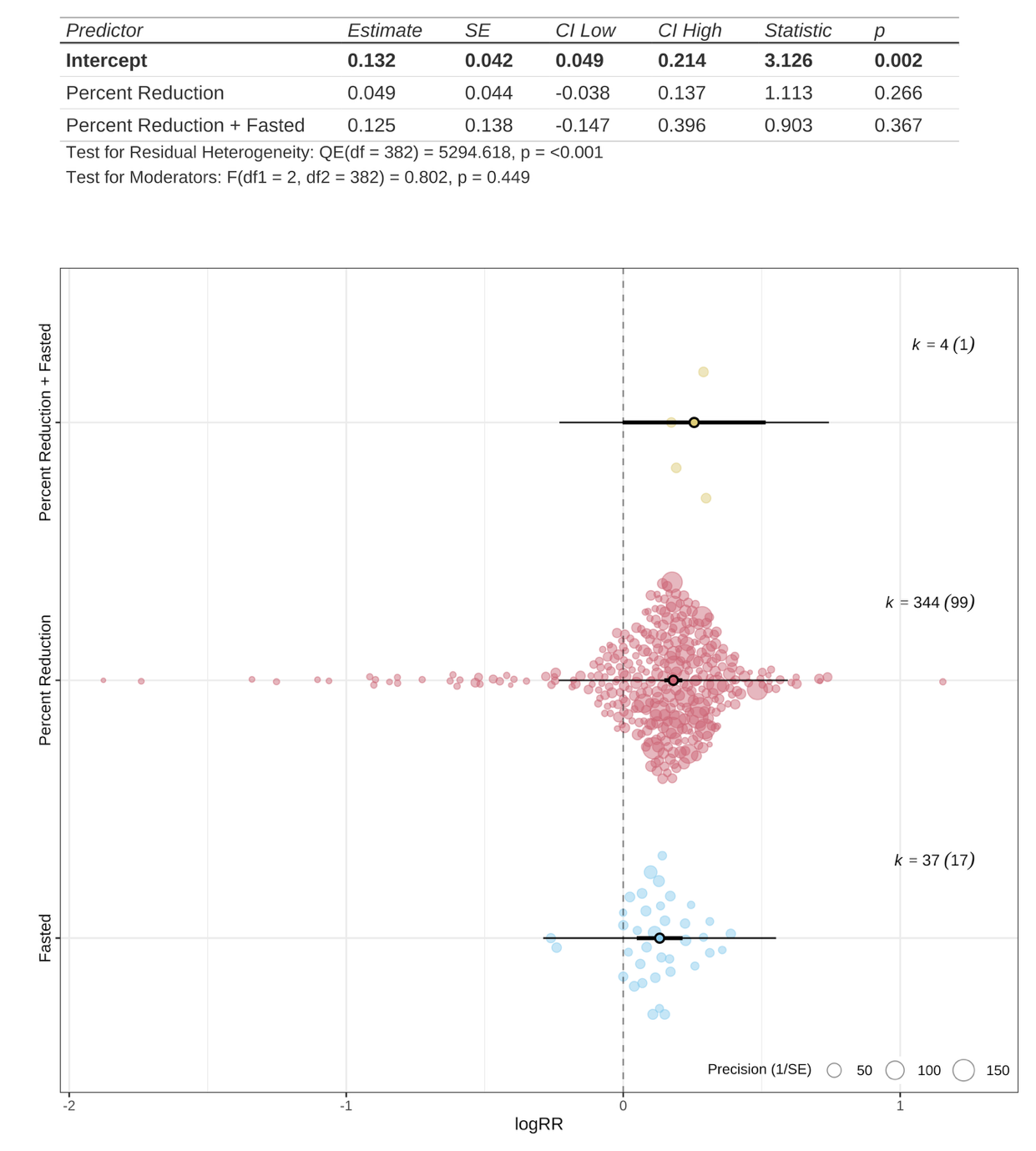

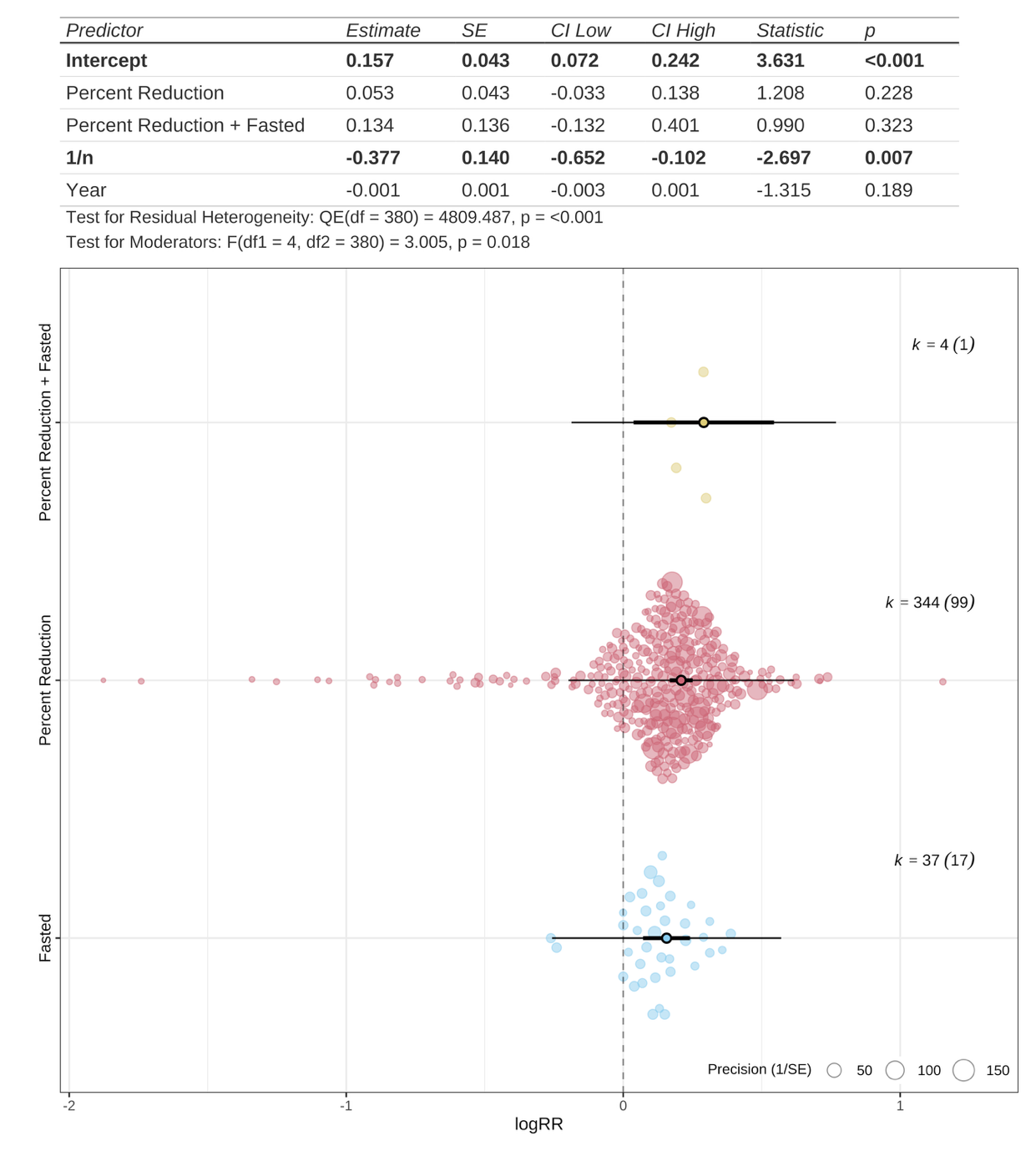


**Figure S14.** Model output from a multi-level model of the effect of sex acting on dietary restriction without (left) and with (right) publication bias correction using median values. Each model is associated with a corresponding model table describing the various predictors, estimates, standard error, low and high 95% confidence intervals, test statistic (*t)*, p value, along with a test for residual heterogeneity and moderators. Bolded rows represent moderators or levels of moderator that are significant (α = 0.050). Below this table is an orchard plot which provides a mean value with surrounding 95% confidence intervals (larger lines) and prediction intervals (thinner lines). The coloured dots represent individua effects sized by precision (1/standard error). The number of effect sizes is given on the right with studies in brackets.


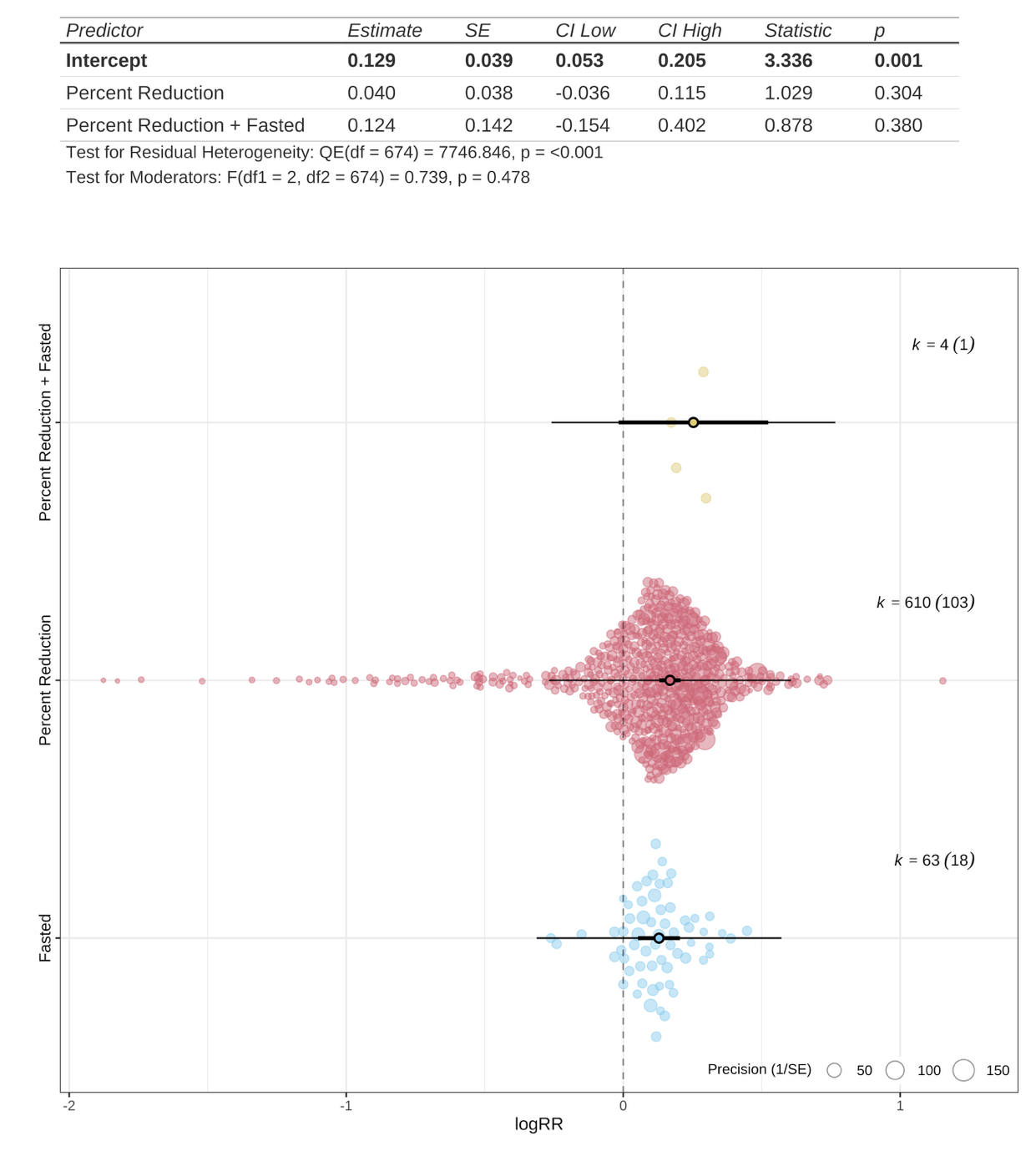

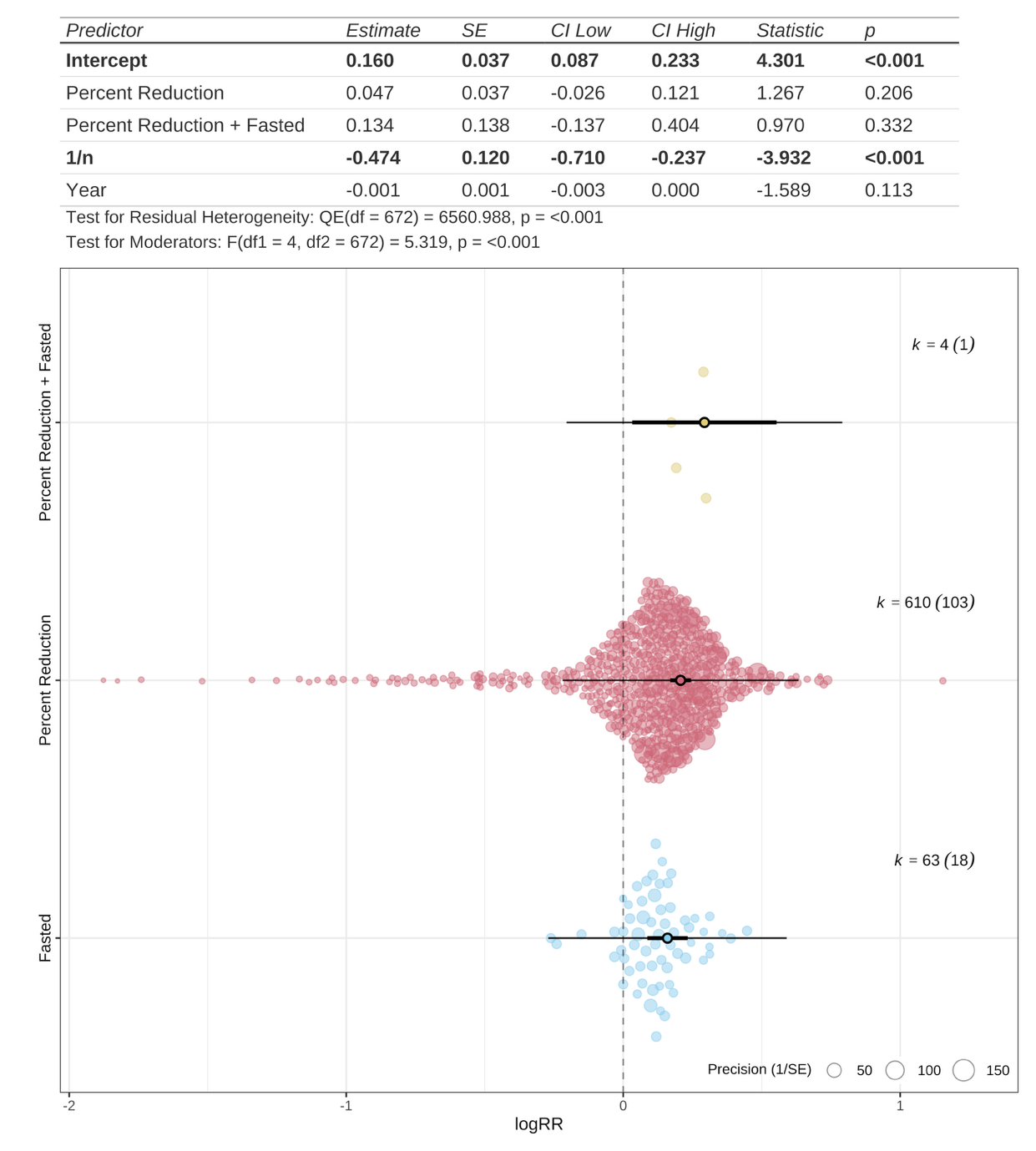


**Figure S15.** Model output from a multi-level model of the effect of dietary restriction methodology without (left) and with (right) publication bias correction using both mean and median values. Each model is associated with a corresponding model table describing the various predictors, estimates, standard error, low and high 95% confidence intervals, test statistic (*t)*, p value, along with a test for residual heterogeneity and moderators. Bolded rows represent moderators or levels of moderator that are significant (α = 0.050). Below this table is an orchard plot which provides a mean value with surrounding 95% confidence intervals (larger lines) and prediction intervals (thinner lines). The coloured dots represent individual effects sized by precision (1/standard error). The number of effect sizes is given on the right with studies in brackets.


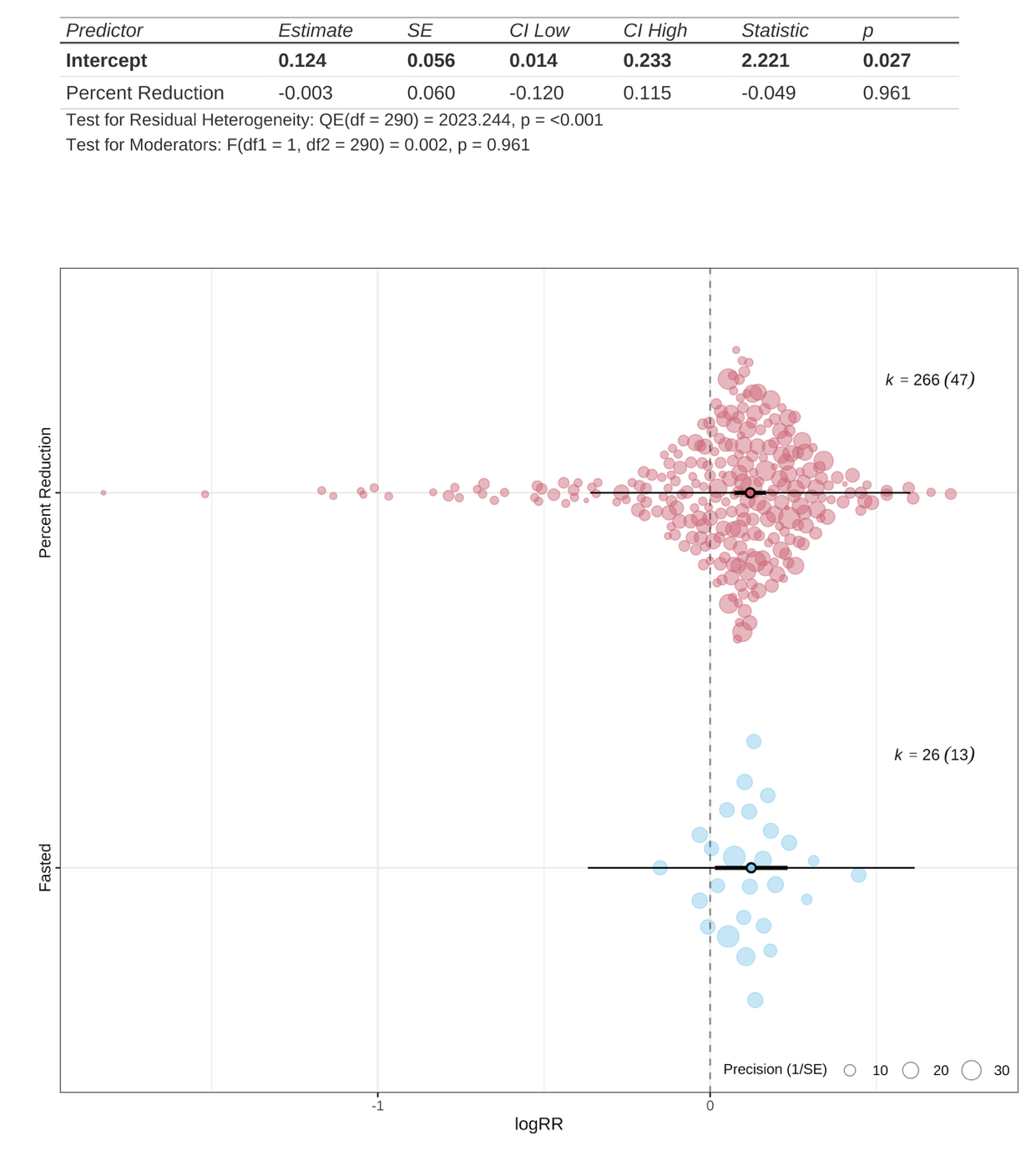

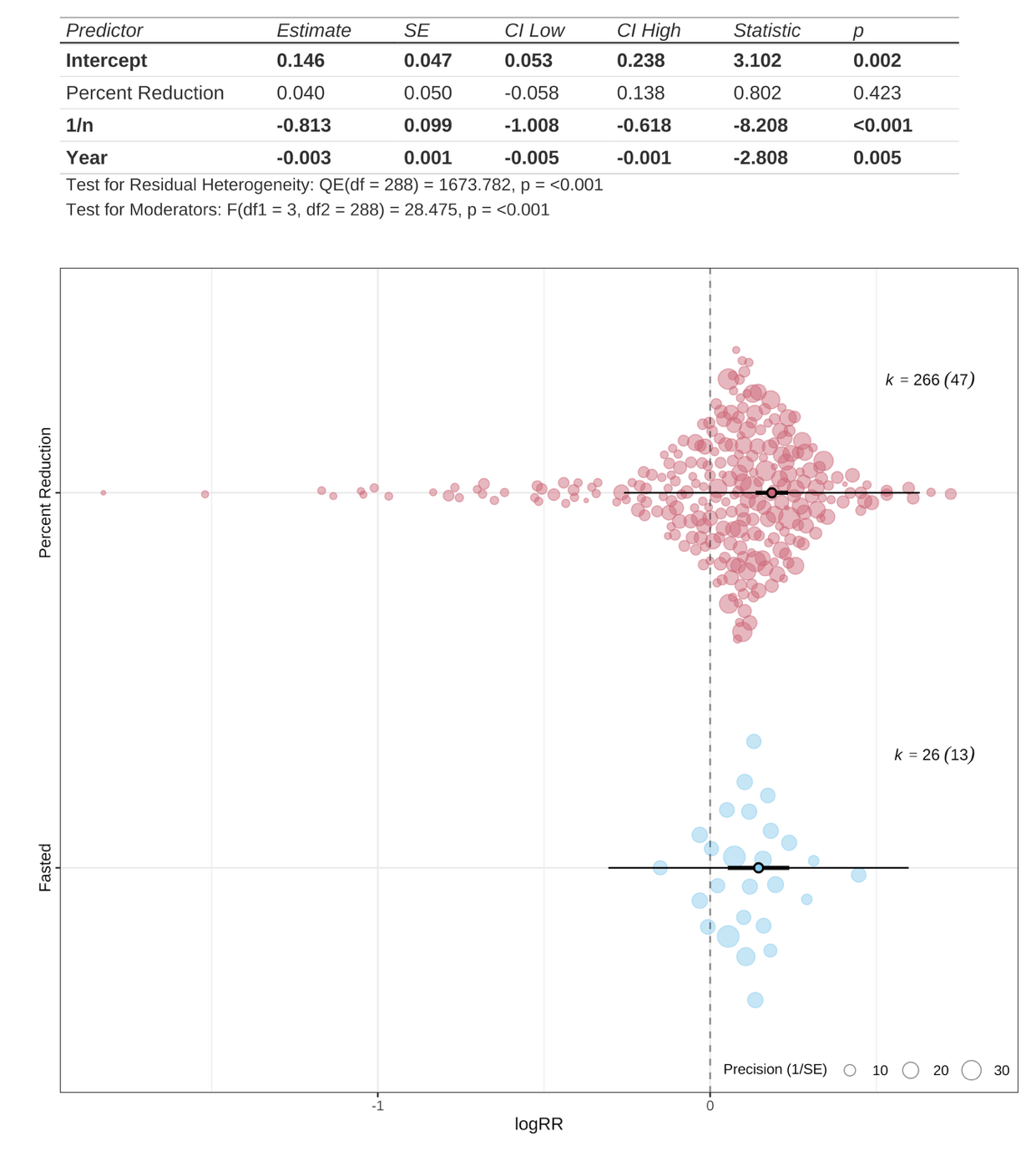


**Figure S16.** Model output from a multi-level model of the effect of dietary restriction methodology without (left) and with (right) publication bias correction using mean values. Each model is associated with a corresponding model table describing the various predictors, estimates, standard error, low and high 95% confidence intervals, test statistic (*t)*, p value, along with a test for residual heterogeneity and moderators. Bolded rows represent moderators or levels of moderator that are significant (α = 0.050). Below this table is an orchard plot which provides a mean value with surrounding 95% confidence intervals (larger lines) and prediction intervals (thinner lines). The coloured dots represent individual effects sized by precision (1/standard error). The number of effect sizes is given on the right with studies in brackets.


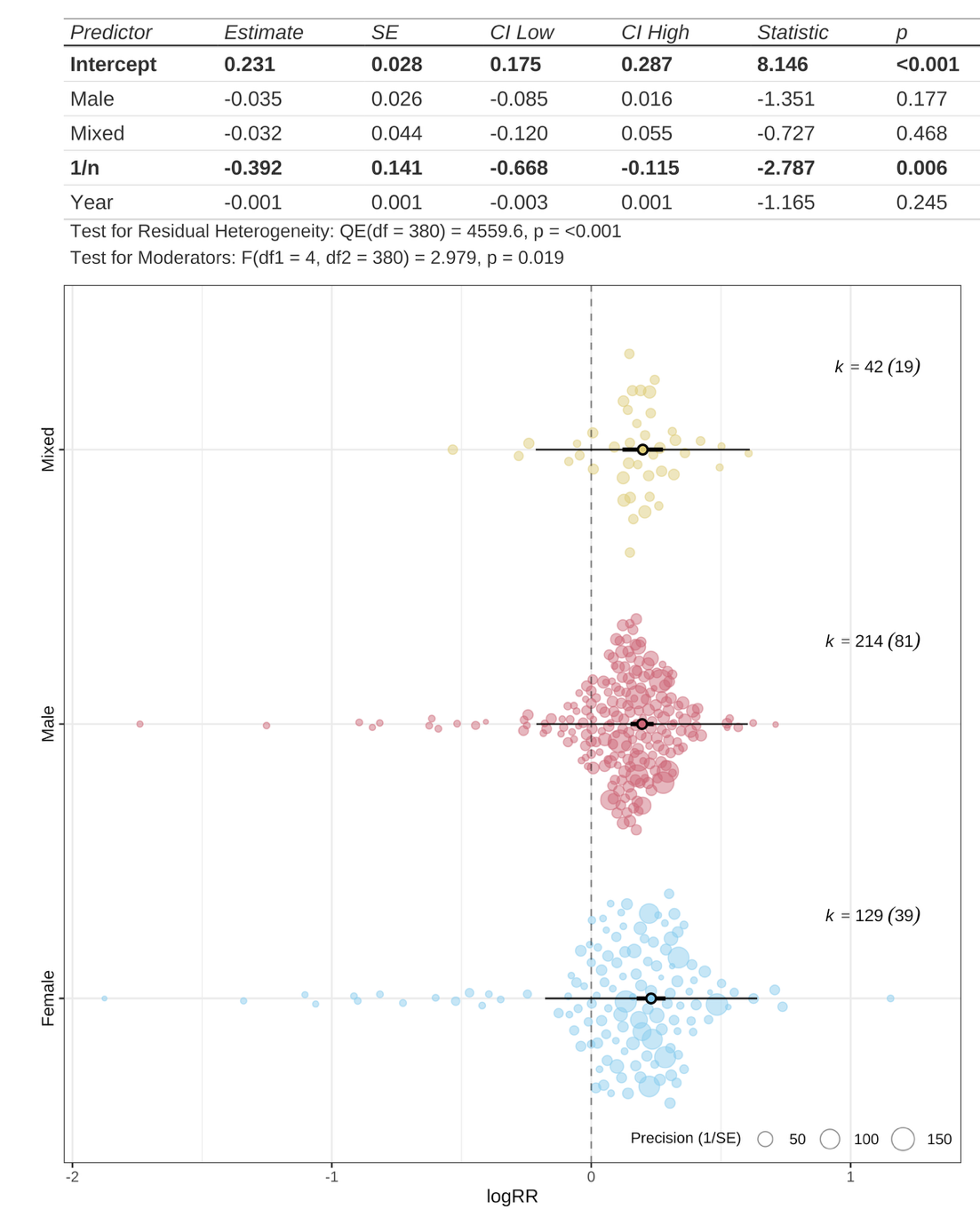

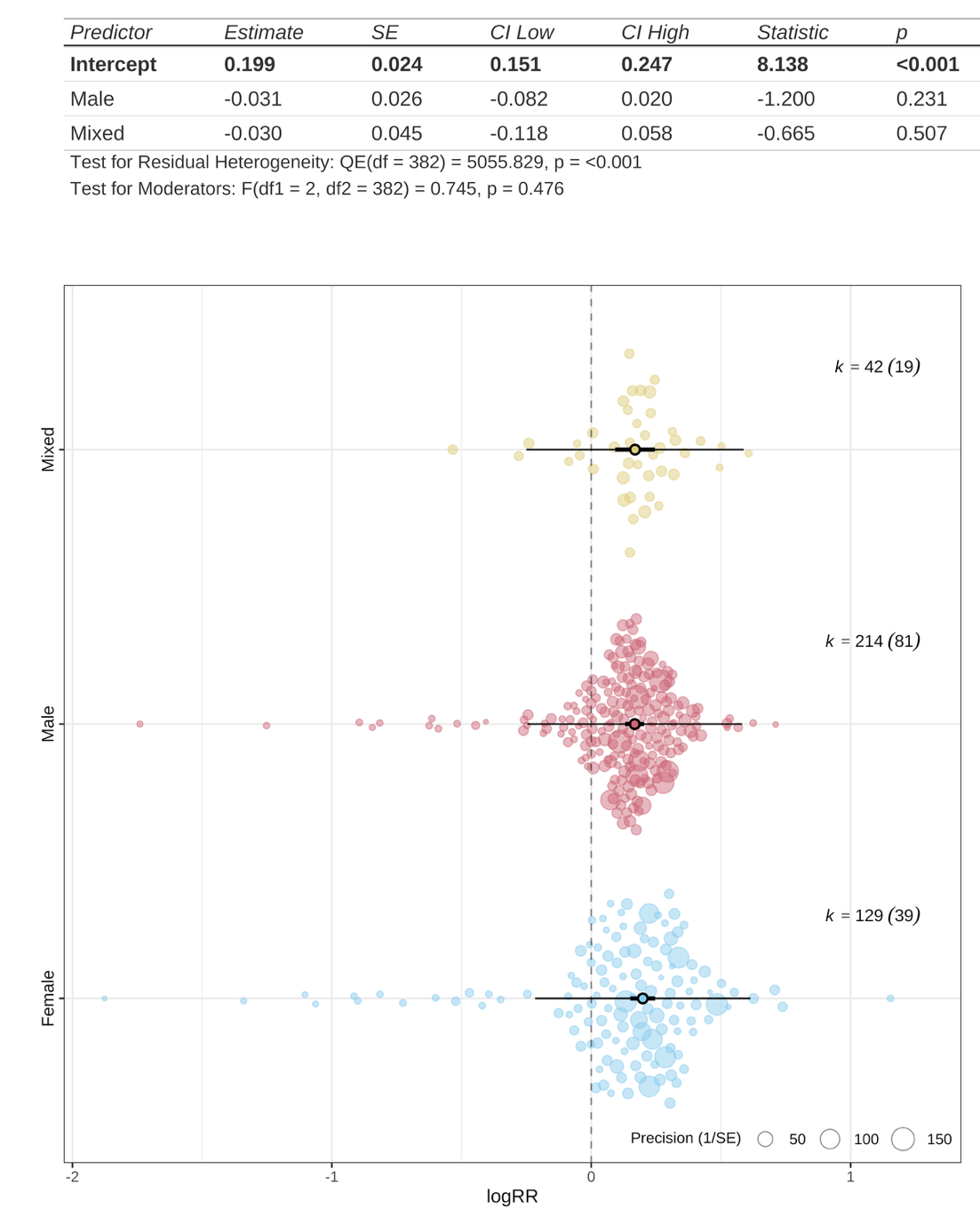


**Figure S17.** Model output from a multi-level model of the effect of dietary restriction methodology without (left) and with (right) publication bias correction using median values. Each model is associated with a corresponding model table describing the various predictors, estimates, standard error, low and high 95% confidence intervals, test statistic (*t)*, p value, along with a test for residual heterogeneity and moderators. Below this table is an orchard plot which provides a mean value with surrounding 95% confidence intervals (larger lines) and prediction intervals (thinner lines). The coloured dots represent individual effects sized by precision (1/standard error). The number of effect sizes is given on the right with studies in brackets.


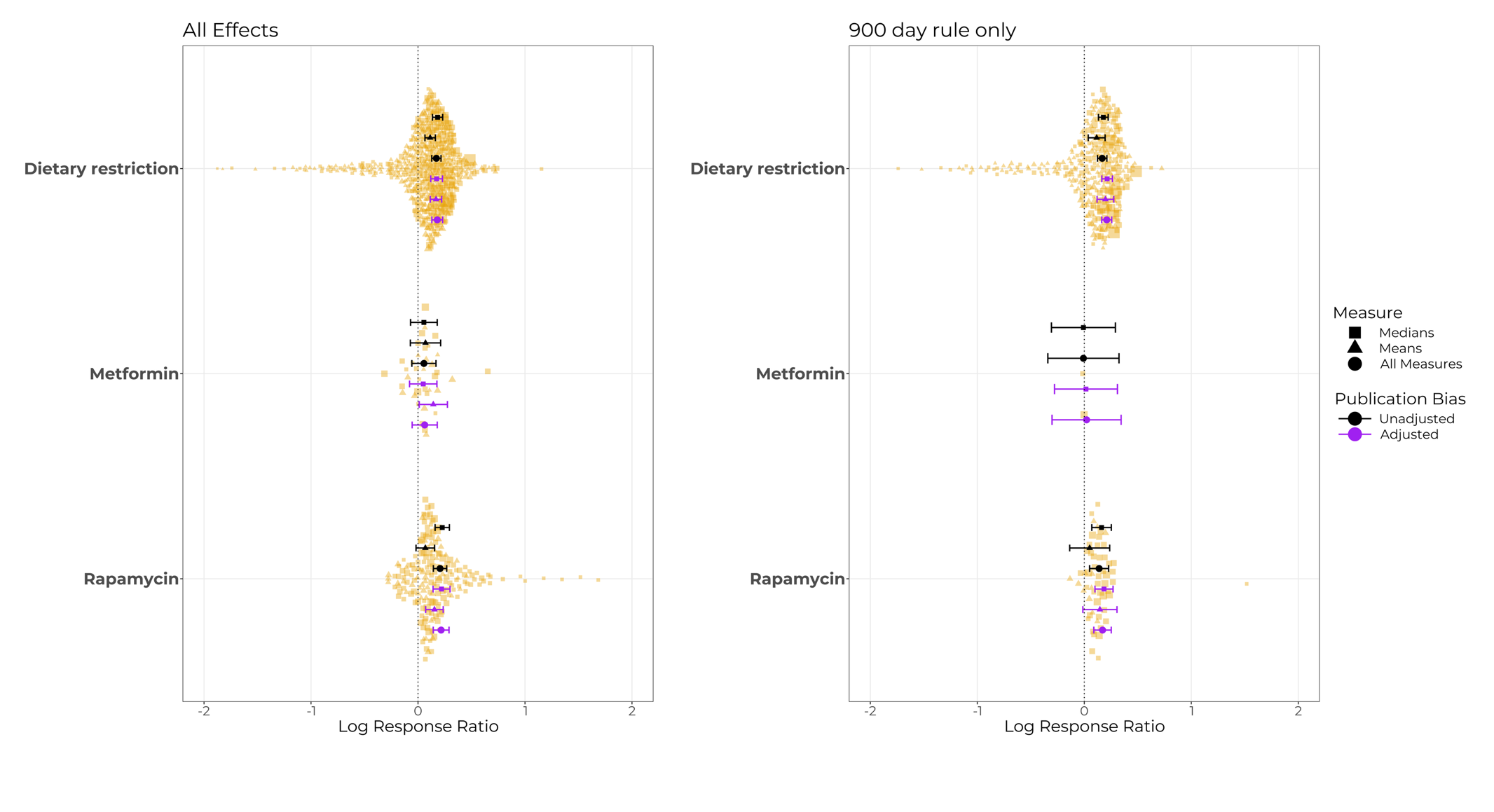
**Figure 18.** The mean effect of dietary restriction, metformin, and rapamycin in mice. Each treatment has a mean effect size with surrounding 95% confidence intervals. A positive mean effect indicates an overall lifespan-extending effect of the treatment, whereas a negative is the opposite. Means and errors are shown from models unadjusted (black) or adjusted (purple) for publication bias, as well as originating from models with only medians (squares), only means (triangles) or using both measures combined (circle). Points represent individual effect sizes scaled by precision (1/standard error), shapes denote measure type and colour denotes species (orange = mice). The panel on the left denotes all effect sizes originating from mice, whereas the panel on the right only contains effect sizes where the control lifespan is greater than 850 days or the lifespan of the intervention group is >950 days (*sensu* Pabis et al. 2024). We note that although this study presents means in addition to medians, the original paper specifically refers to median lifespan.

***References***

Pabis, K., D. Barardo, J. Gruber, O. Sirbu, M. Malavolta, K. Selvarajoo, M. Kaeberlein, et al. 2024. The impact of short-lived controls on the interpretation of lifespan experiments and progress in geroscience – Through the lens of the “900-day rule.” Ageing Research Reviews 101:102512.
